# Similarities and differences of a proliferation-inducing ligand expression in lacrimal gland lesions of patients with IgG4-associated ophthalmic diseases and mucosa-associated lymphoid tissue lymphoma

**DOI:** 10.3389/fimmu.2025.1514003

**Published:** 2025-02-18

**Authors:** Lvfu He, Lisha Zhan, Yu Yang, Weimin He

**Affiliations:** ^1^ Department of Ophthalmology, West China Hospital of Sichuan University, Chengdu, Sichuan, China; ^2^ The Third Hospital of Mianyang, Sichuan Mental Health Center, Mianyang, Sichuan, China; ^3^ Department of Ophthalmology, Sichuan Academy of Medical Sciences & Sichuan Provincial People’s Hospital, University of Electronic Science and Technology of China, Chengdu, Sichuan, China

**Keywords:** lacrimal gland, mucosa-associated lymphoid tissue (MALT) lymphoma, IgG4-related ophthalmic disease (IgG4-ROD), a proliferation inducing ligand (APRIL), immunoglobulin G4 (IgG4)

## Abstract

**Objective:**

This study aimed to investigate the expression condition of a proliferation-inducing ligand (APRIL) in lacrimal gland lesions of patients with IgG4-associated ophthalmic diseases (IgG4-ROD) and mucosa-associated lymphoid tissue (MALT) lymphoma.

**Patients and methods:**

Fifteen patients with IgG4-ROD, 3 with MALT lymphoma, and 1 with elevated IgG4 with lacrimal gland lesions, treated in West China Hospital of Sichuan University from April 2022 to November 2023, were included. Immunofluorescence staining was used to detect the expression of APRIL in the specimen of lacrimal gland.

**Results:**

The average expression level of APRIL in patients with lacrimal gland lesions of IgG4-ROD and MALT lymphoma were 8471.12 pixels/HPF and 2950.78 pixels/HPF respectively. The positive rates of APRIL were 10.49% and 7.23% respectively. CD138 and APRIL were colocalized, and the positive rate of their colocalization was 8.83%, and the positive areas of colocalization coincidence was 946.84 pixels/HPF in patients with IgG4-ROD. CD20 and APRIL were colocalized, and the positive rate of their colocalization was 7.04%, and the positive areas of colocalization coincidence was 949.78 pixels/HPF in patients with MALT lymphoma. We also found that the expression level and the positive rate of APRIL were positively correlated with the level of serum IgG4 in IgG4-ROD patients (r=0.5820, *P*=0.029; r= 0.6261, *P*=0.017; respectively). In addition, the positive rate and the positive areas of CD138 and APRIL colocalization were also positively correlated with serum IgG4 level (r=0.6420, *P*=0.013; r= 0.5673, *P*=0.034; respectively).

**Conclusion:**

APRIL is highly expressed in lacrimal gland lesions of patients with IgG4-ROD and MALT lymphoma. This overexpression may facilitate the enrichment of CD138^+^ plasma cells and is associated with elevated serum IgG4 levels in patients with IgG4-ROD. Additionally, it may promote the proliferation of CD20^+^ B lymphocytes in patients with MALT lymphoma.APRIL may play a certain role in the possible transformation of IgG4-ROD into MALT lymphoma.

## 1 Introduction

Immunoglobulin G4-related disease (IgG4-RD) is a complex immune-mediated inflammatory condition that has the potency to impact numerous organ systems (1). Specifically, when the eye is affected, it is called immunoglobulin G4-related ophthalmic disease (IgG4-ROD). Numerous studies confirmed the involvement of T cell subsets (2–4), plasma blast cells (5), B cells (6), macrophages (7), eosinophils (8) and mast cells (9) in the pathological processes of IgG4-RD. The disease was characterized by the activation of type 2 helper T cells and regulatory T cells, leading to the production of cytokines that promote fibrosis and an increased synthesis of Immunoglobulin G4 (IgG4) and IgE (10). The main pathological features of IgG4-RD include infiltration of IgG4^+^ plasma cells, tumor-like growth, matted fibrosis, and obliterative phlebitis (11). While occlusive phlebitis is rare in IgG4-ROD patients (12), collagen fibrosis is commonly observed in lacrimal gland lesions (11, 13).

A proliferation-inducing ligand (APRIL) is expressed in various immune cells, including neutrophils, eosinophils, and macrophages (14–16). It serves several physiological functions, such as inducing immunoglobulin class switching and promoting the survival of plasma cells (17). These effects are mediated through two distinct APRIL signaling receptor pathways: transmembrane activator and calcium regulator with cyclophilin coupling (TACI) and B cell maturation antigen (BCMA) (18). Additionally, APRIL requires binding to receptors for heparan sulfate proteoglycans (HSPG) to effectively transmit signals to plasma cells (19, 20). Moreover, studies found that APRIL was highly expressed in submandibular gland and renal tissue lesions in IgG4-RD patients, which may act on CD138^+^ plasma cells to promote the enrichment of CD138 ^+^ plasma cells (21). And APRIL increased in gastric MALT lymphoma, which may act on CD20^+^ B lymphocytes to promote CD20^+^ B lymphocyte proliferation (22). IgG4-RD could the potential to evolve into MALT lymphoma (23, 24). The association of IgG4-RD with MALT lymphoma has also been gradually noted and studied.

Therefore, we conducted this trial to investigate the expression and function of APRIL in lacrimal gland lesions of IgG4-ROD and MALT lymphoma.

## 2 Patients and methods

### 2.1 Patients information

We included lacrimal gland specimens from 15 cases of IgG4-ROD, 3 cases of MALT lymphoma, and 1 case of MALT lymphoma with elevated IgG4 levels, all of which were diagnosed through immunohistochemistry and molecular pathology. All patients underwent lacrimal gland lesion resection at West China Hospital of Sichuan University from April 2022 to November 2023. Clinical data and fasting serum IgG4 levels in the morning before treatment were also collected. This study was approved by the ethics committee of our hospital (Approval Number: 2022 Review 17789), and all patients provided informed consent.

IgG4-RD were diagnosed by the Japanese Study Group in 2014, as following: (1) imaging examination showed lacrimal gland enlargement; (2) histopathological characteristics are consistent with IgG4-RD; and (3) elevated serum IgG4 (25). According to these criteria, IgG4-RD was classified as definitive, probable and possible when patient fulfilled all three criteria, the first two criteria and the first and last criteria, respectively. In our study, definite and probable categories were taken to be IgG4-RD.

### 2.2 Paraffin tissue preparation

All the freshly obtained lacrimal tissue was processed into paraffin-embedded tissue, following a precise protocol. The detailed steps are outlined below: 1. After a 24-hour fixation period in a universal tissue fixation solution, the tissue was thoroughly rinsed with pure water. 2.Dehydration was achieved through sequential immersion in 70%, 80%, and 95% alcohol solutions, followed by anhydrous alcohol, with each step lasting for 2 hours. 3. The tissue sample was then purified using a 1:1 mixture of anhydrous alcohol and xylene. Subsequently, it was placed in xylene I and xylene II for 2 hours each, facilitating further clearing. 4. The transparently processed samples were individually immersed in three wax cups (labeled I, II, III) within a wax melting box maintained at 58°C. Each cup was utilized for 2 hours to ensure thorough wax infiltration. 5. Once fully impregnated with wax, the sample was positioned in melted pure paraffin for meticulous embedding. 6. Utilizing a paraffin microtome, the paraffin-embedded tissue was meticulously sectioned into 4-micron slices, ready for subsequent staining procedures.

### 2.3 Double immunofluorescence staining

For the IgG4-ROD lacrimal gland paraffin sections, CD138 and Aprily-8 double immunofluorescence staining was employed, while CD20 and Aprily-8 double immunofluorescence staining was used for the MALT lymphoma lacrimal gland paraffin sections. The specific steps are as follows:1.Dewaxing in a graded series of ethanol.2.Antigen retrieval was performed using pH 8.0 EDTA, followed by blocking the tissue sections with 3% BSA for 30 minutes.3.A mixture of the primary monoclonal rabbit antibody CD138 (1:500, Abcam, UK) or CD20 (1:10, Abcam, UK) and the second primary monoclonal mouse antibody Aprily-8 (1:100, Novus Biologicals, USA) was added and incubated overnight in a refrigerator at 4°C.4.The corresponding secondary antibody for CD138 or CD20 (Cy3-labeled goat anti-rabbit IgG) and the secondary antibody for Aprily-8 (Alexa Fluor 488-labeled goat anti-mouse IgG) were added sequentially, incubating them in a 37°C incubator for 50 minutes, followed by several washes.5.The cell nuclei were re-stained with DAPI, and dropping tissue autofluorescence quencher (Servicebio, China), and the slides were then mounted.6.Each pathological section was photographed under an fluorescence microscope (Nikon, Japan), capturing three fields at ×200 magnification and three fields at ×400 magnification. CD138, CD20 cells, and APRIL areas were quantified using Aipathwell software (Servicebio, China).

### 2.4 Triple immunofluorescence staining

Paraffin sections of MALT lymphoma lacrimal glands with elevated IgG4 levels were subjected to CD20, CD138, and Aprily-8 triple immunofluorescence staining. The specific steps are as follows:1.Dewaxing in a graded series of ethanol.2.Antigen retrieval using pH 6.0 citrate buffer, and the endogenous peroxidase was blocked by incubation with 3%H2O2 at room temperature for 25 min.3.Blocking the sections with serum at room temperature for 30 minutes.4.Adding the primary monoclonal rabbit antibody CD20 (1:1000) and incubating overnight in a refrigerator at 4°C.5.Adding the corresponding secondary antibody (HRP-labeled goat anti-rabbit IgG), followed by incubation in a 37°C incubator for 50 minutes and several washes.6. Adding 50μL TSA (Tyramide signal amplification) (Servicebio, China)-555 dyeing solution to ensure complete tissue coverage, and incubate at room temperature for 10 min.7. Placing the tissue sections in a repair box filled with pH 6.0 citric acid repair solution (G1202) and heating in a microwave for 10 minutes. 8. Blocking the sections with serum at room temperature for 30 minutes. 9. Adding the second primary monoclonal rabbit antibody CD138 (1:5000) and incubating overnight in a refrigerator at 4°C.10. Adding the corresponding secondary antibody, followed by incubation in a 37°C incubator for 50 minutes and several washes.11. Adding 50μL TSA-488 dyeing solution to ensure complete tissue coverage, and incubate at room temperature for 10 min.12. Placing the tissue sections in a repair box filled with pH 6.0 citric acid repair solution and heating in a microwave for 10 minutes. 13. Blocking the sections with serum at room temperature for 30 minutes. 14.Adding the third primary monoclonal rabbit antibody Aprily-8 (1:1000) and incubating overnight in a refrigerator at 4°C.15.Adding the corresponding secondary antibody, followed by incubation in a 37°C incubator for 50 minutes and several washes.16. Adding 50μL TSA-647 dyeing solution to ensure complete tissue coverage, and incubate at room temperature for 10 min.17.Re-staining the cell nuclei with DAPI, and dropping tissue autofluorescence quencher, and finally mounting the slides.18.Each pathological section was photographed under an fluorescence microscope (Nikon, Japan), capturing three fields at ×200 magnification and three fields at ×400 magnification. CD138, CD20 cells, and APRIL areas were quantified using Aipathwell software (Servicebio, China).

### 2.5 Index of immunofluorescence staining

The immunofluorescence staining indexes recorded in this study are as follows: Red positive cells = total number of red positive cells in tissue area; Green positive area = green positive area in tissue area; Red positive rate = total number of red positive cells/total number of cells; Green positive rate = total green positive area/total area; Positive rate of colocalization = total number of colocalization/(total number of red positive cells + total area of green positive cells - total number of colocalization);Colocalization coincidence positive area = colocalization red and green coincidence yellow positive pixel area.

### 2.6 Statistic Analysis

Continuous variables are presented as mean ± standard deviation. Pearson correlation analysis was employed to assess the relationship between CD138, APRIL, and serum IgG4 levels. A p-value of < 0.05 was considered statistically significant. Statistical analyses were performed using GraphPad Prism 8.0 (GraphPad, USA).

## 3 Results

### 3.1 Demographic characteristics

The mean age of patients with IgG4-ROD was 47.0 years, while that of patients with MALT lymphoma was 56.8 years. Among the IgG4-ROD patients, 60% were male, whereas 75% of MALT lymphoma patients were female. The mean duration of symptoms was 42.4 months for IgG4-ROD and 63.0 months for MALT (**Table 1**).

**Table 1:** Demographic characteristics of patients with IgG4-ROD and MALT lymphoma.

	IgG4-ROD	MALT lymphoma
Number	15	4
Age, mean ± standard deviation (range)	47.0 ± 14.4 (28-68)	56.8 ± 18.4 (39-82)
Sex		
Male, number (%)	9 (60.0%)	1 (25.0%)
Female, number (%)	6 (40.0%)	3 (75.0%)
Laterality		
Right, number (%)	7 (46.7%)	2 (50.0%)
Left, number (%)	8 (53.3%)	2 (50.0%)
Symptom presenting period, mean ± standard deviation (range)	42.4 ± 22.6 (12-96)	63.0 ± 45.3 (12-120)
Allergy history, number (%)	1 (6.7%)	1 (25.0%)

IgG4-ROD, Immunoglobulin G4-related ophthalmic disease; MALT, Mucosa-associated lymphoid tissue.

### 3.2 Expression of APRIL in lacrimal gland lesions of IgG4-ROD and MALT lymphoma patients

**Figure 1:**
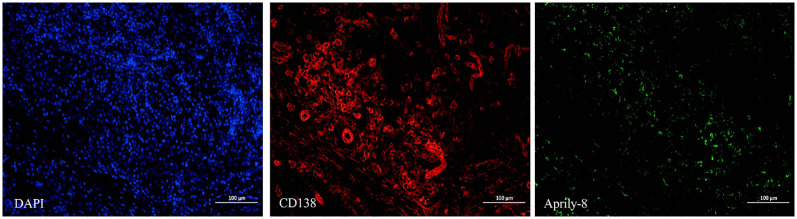
CD138 (red) and Aprily-8 immunofluorescence staining secreted APRIL (green) in lacrimal gland lesions of IgG4-ROD patients (×200). APRIL, A proliferation-inducing ligand; IgG4-ROD, Immunoglobulin G4-related ophthalmic disease.

**Figure 2:**
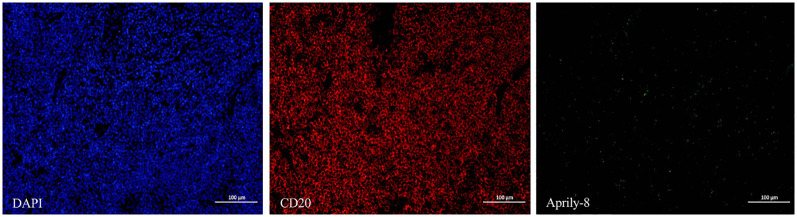
CD20 (red) and Aprily-8 immunofluorescence staining secreted APRIL (green) in lacrimal gland lesions of MALT lymphoma patients (×200). APRIL, A proliferation-inducing ligand; MALT, Mucosa-associated lymphoid tissue.

In the lacrimal gland lesions of IgG4-ROD patients, CD138 and secreted APRIL were highly expressed, with a mean of 1152.51 cells/HPF (range: 138.00–2678.00 cells/HPF) and a mean of 8471.12 pixels/HPF (range: 8.00–92794.00 pixels/HPF) (**Figure 1**). Notably, the positive rate for CD138 was 35.59% (range: 3.30–93.46%), while the positive rate for APRIL was 10.49% (range: 0.03–56.40%). In contrast, lacrimal gland lesions in MALT lymphoma patients exhibited high expression of CD20 and secreted APRIL, with a mean of 4109.44 cells/HPF (range: 2905.00–4927.00 cells/HPF) and a mean of 2950.78 pixels/HPF (range: 699.00–4541.00 pixels/HPF) (**Figure 2**). The positive rate for CD20 was 85.48% (range: 71.18–96.42%), while the positive rate for APRIL was 7.23% (range: 1.98–12.22%) (**Table 2**).

**Table 2:** Expressions of CD138 and APRIL, and CD20 and APRIL in lacrimal gland lesions in IgG4-ROD and MALT lymphoma patients, respectively.

		IgG4-ROD	MALT lymphoma
CD138	cells	1152.51 ± 698.31	
	the positive rate	35.59 ± 22.72	
CD20	cells		4109.44 ± 610.98
	the positive rate		85.48 ± 8.78
APRIL	areas	8471.12 ± 18700.94	2950.78 ± 1591.97
	the positive rate	10.49 ± 13.25	7.23 ± 3.98

All results expressed as: mean ± standard deviation.

APRIL, A proliferation-inducing ligand; IgG4-ROD, Immunoglobulin G4-related ophthalmic disease; MALT, Mucosa-associated lymphoid tissue.

### 3.3 Target cells of APRIL in lacrimal gland lesions of patients with IgG4-ROD and MALT lymphoma

**Figure 3:**
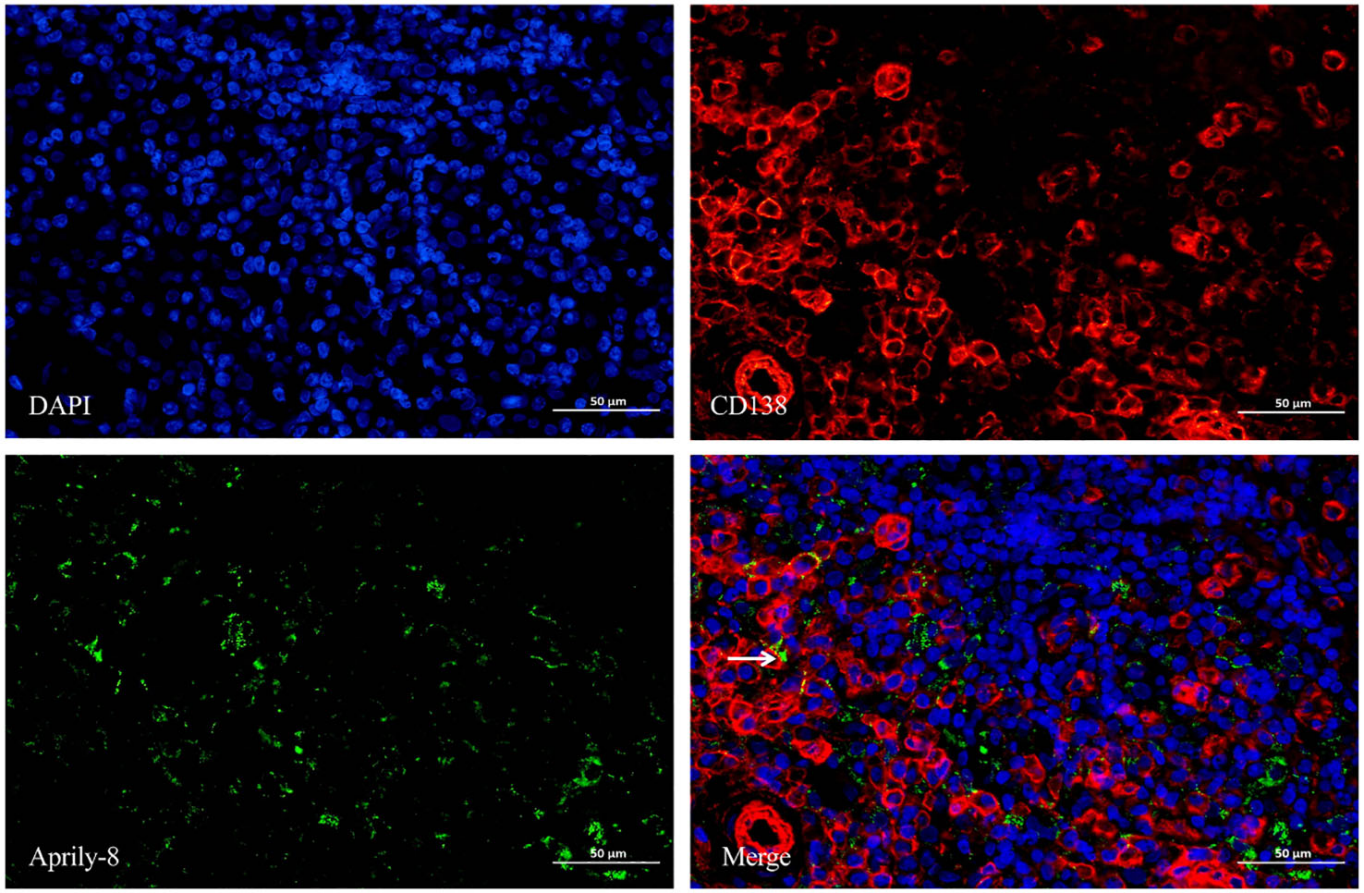
Double immunofluorescence staining (×400) of CD138 (red) and Aprily-8 (green) in lacrimal gland lesions of IgG4-ROD patients. White arrows show the co-localization of CD138 and secreted APRIL. IgG4-ROD, Immunoglobulin G4-related ophthalmic disease; APRIL, A proliferation-inducing ligand.

**Figure 4:**
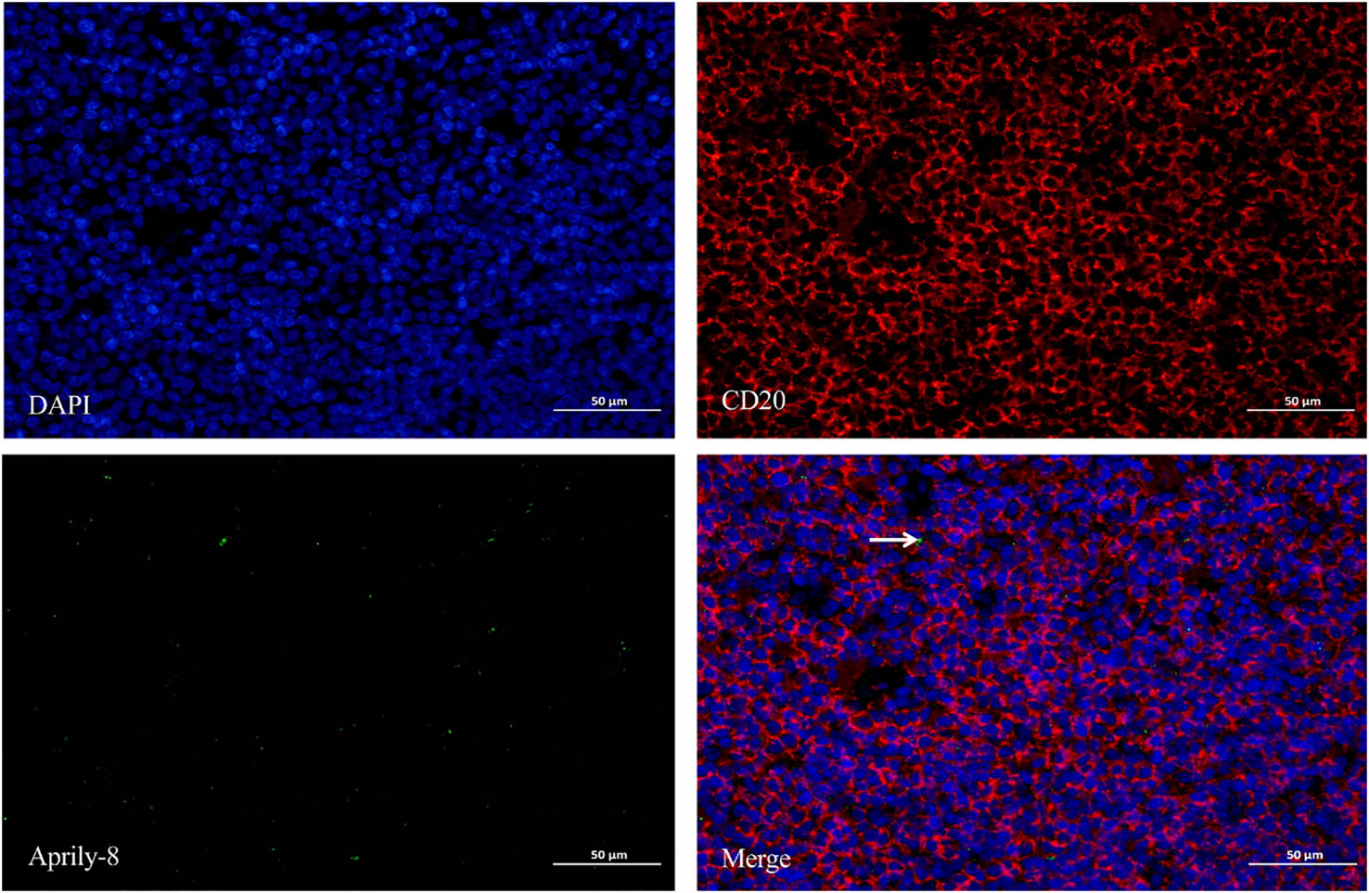
Double immunofluorescence staining (×400) of CD20 (red) and Aprily-8 (green) in lacrimal gland lesions in MALT lymphoma patients, with white arrows showing the co-localization of CD20 and secretory APRIL. MALT, Mucosa-associated lymphoid tissue; APRIL, A proliferation-inducing ligand.

Dual immunofluorescence staining revealed colocalization of CD138 and APRIL in lacrimal gland lesions of patients with IgG4-ROD (**Figure 3**). The positive rate for the co-localization of CD138 and APRIL was 8.83% (range: 0.15–45.44%), with the colocalization coincidence positive areas measuring 946.84 pixels/HPF (range: 3.00–7788.00 pixels/HPF). This indicates that secreted APRIL was primarily distributed near or on the infiltrated CD138^+^ plasma cells. In lacrimal gland lesions of MALT lymphoma patients, CD20 and APRIL were also found to be co-localized (**Figure 4**, **Table 3**). The positive rate for the co-localization of CD20 and APRIL was 7.04% (range: 1.27–12.51%), with colocalization coincidence positive areas of 949.78 pixels/HPF (range: 160.00–2122.00 pixels/HPF). This suggests that secreted APRIL is predominantly found near or on the infiltrated CD20^+^ plasma cells.

**Table 3:** Co-expression of CD138 and APRIL, and CD20 and APRIL in lacrimal gland lesions in IgG4-ROD and MALT lymphoma patients, respectively.

	positive rate of their colocalization	positive area of colocalization coincidence
IgG4-ROD	8.83 ± 11.01	946.84 ± 1864.70
MALT lymphoma	7.04 ± 4.17	949.78 ± 688.32

All results expressed as: mean ± standard deviation.

APRIL, A proliferation-inducing ligand; IgG4-ROD, Immunoglobulin G4-related ophthalmic disease; MALT, Mucosa-associated lymphoid tissue.

### 3.4 Expression of APRIL in lacrimal gland lesions of MALT lymphoma patients with elevated IgG4

**Figure 5:**
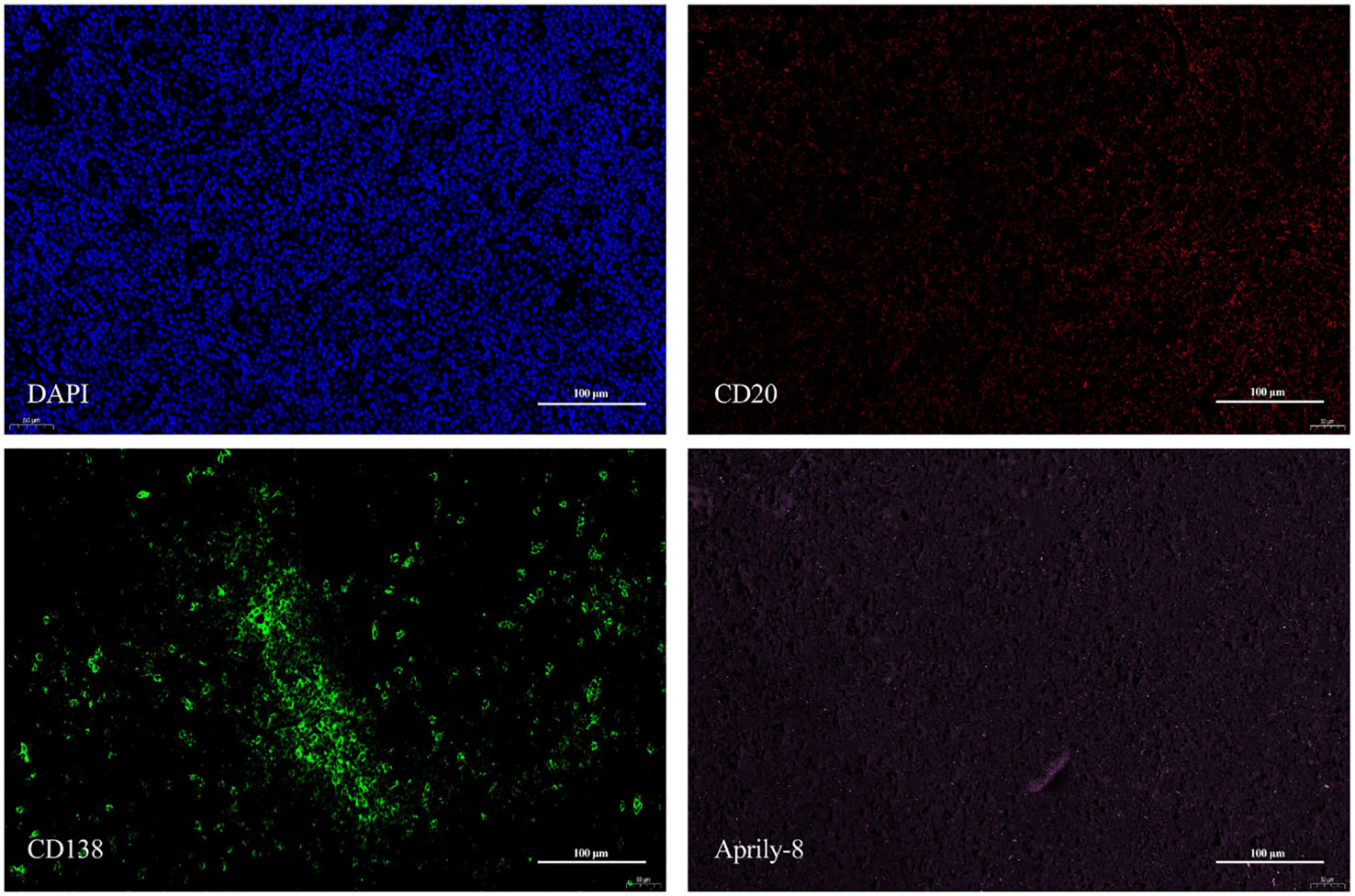
CD20 (red), CD138 (green), and Aprily-8 (pink) immunofluorescence staining (×200) of lacrimal gland lesions of MALT Lymphoma patients with elevated IgG4. MALT, Mucosa-associated lymphoid tissue; IgG4, Immunoglobulin G4.

**Figure 6:**
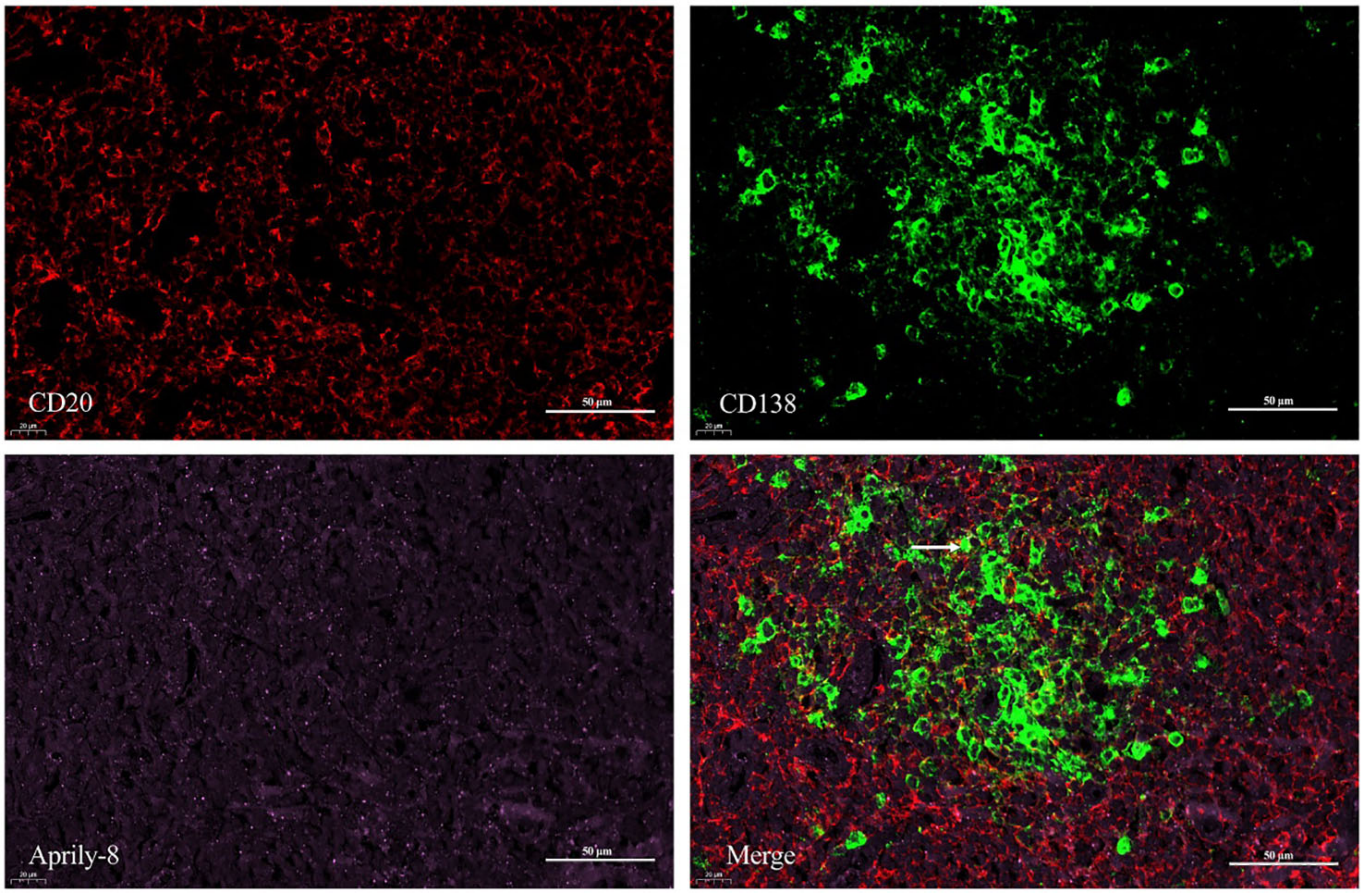
Triple immunofluorescence staining (×400) of CD20 (red), CD138 (green), and Aprily-8 (pink) in lacrimal gland lesions of MALT lymphoma patients with elevated IgG4. White arrows show the co-localization of CD20, CD138, and secreted APRIL. MALT, Mucosa-associated lymphoid tissue; IgG4, Immunoglobulin G4.

One patient with MALT lymphoma had a serum IgG4 level of 4.55g/L. In the patient’s lacrimal gland lesions, We observed high expression levels of CD20, CD138, and secreted APRIL. Specifically, the mean counts were 7436.67 cells/HPF (range: 6538.00–8060.00 cells/HPF) for CD20, 547.67 cells/HPF (range: 126.00–954.00 cells/HPF) for CD138, and an mean area of 1,428,066.33 pixels/HPF (range: 1,320,610.00–1,521,975.00 pixels/HPF) for secreted APRIL (**Figure 5**). Notably, triple immunofluorescence staining demonstrated the colocalization of CD20, CD138, and secreted APRIL in lacrimal gland lesions of MALT lymphoma patients with elevated IgG4 (**Figure 6**).

### 3.5 Relationship between CD138, APRIL and serum IgG4 in lacrimal gland lesions of IgG4-ROD patients

**Figure 7:**
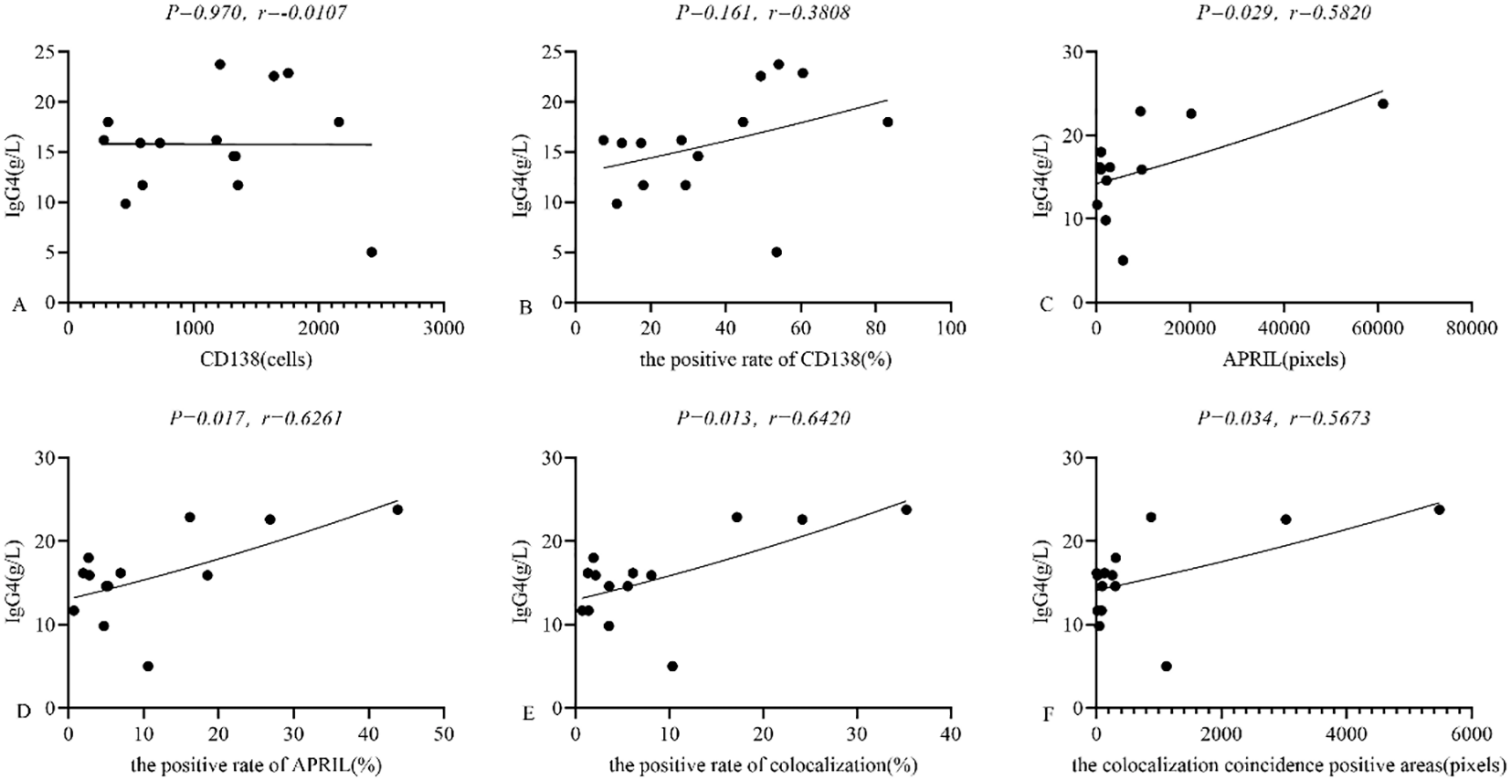
**(A-F)** Relationship between CD138, APRIL and serum IgG4 in lacrimal gland lesions in IgG4-ROD patients. APRIL, A proliferation-inducing ligand; IgG4, Immunoglobulin G4; IgG4-ROD, Immunoglobulin G4-related ophthalmic disease.

In IgG4-ROD patients, serum IgG4 levels averaged 15.64 ± 5.22 g/L (range: 5.00 g/L - 23.79 g/L). In lacrimal gland lesions of these patients, we found that the expression of CD138 and the positive rate of CD138 were not related to the level of serum IgG4(95% CI: -0.52 to 0.50, r = -0.0107, *P* = 0.970; 95% CI: -0.16 to 0.75, r = 0.3808, *P* = 0.161; respectively) (**Figures 7A, B**). In contrast, the expression of secreted APRIL and the positive rate of secreted APRIL in lacrimal gland lesions showed a positive correlation with serum IgG4 levels (95% CI: 0.07 to 0.85, r = 0.5820, *P* = 0.029; 95% CI: 0.14 to 0.87, r = 0.6261, *P* = 0.017; respectively) (**Figures 7C, D**). Furthermore, the positive rate of colocalization and the colocalization coincidence positive areas were positively correlated with serum IgG4 levels (95% CI: 0.17 to 0.87, r = 0.6420, *P* = 0.013; 95% CI: 0.05 to 0.84, r = 0.5673, *P* = 0.034; respectively) (**Figures 7E, F**).

## 4 Discussion

Numerous previous studies found that the increased expression of interleukin-4 (IL-4) and IL-10, secreted by infiltration-induced, costimulatory molecule-positive regulatory T cells (26), M2 macrophages (7), and mast cells (27), may be associated with the immunoglobulin class switch to IgG4. Additionally, APRIL induced IgG4 and IgE class switching in the presence of IL-4 *in vitro* (28). However, the expression of APRIL in lacrimal gland lesions among patients with IgG4-ROD and MALT lymphoma has not been previously reported. Therefore, we investigated the expression of APRIL in these lesions using double and triple immunofluorescence staining.

In this study, we discovered a significant amount of secreted APRIL in lacrimal gland lesions of patients with IgG4-ROD. Antibodies against the extracellular portion of APRIL (amino acids 93-233) were released following proteolytic cleavage, allowing for the identification of APRIL cytokine target cells (29). We further observed that APRIL acted on certain CD138^+^ plasma cells, consistent with findings by Kawakami et al. (21). These results suggested that APRIL may be directly involved in the aggregation of plasma cells in IgG4-ROD lacrimal gland lesions. First of all, APRIL may bind to the BCMA and TACI receptors to trigger a variety of signaling pathways, such as the activation of cysteine-containing aspartic proteases or mitogen-activated protein kinases (MAPK) or c-Jun N-terminal kinase (JNK) or extracellular signal-regulated kinase (ERK), NF-κB translocation (30–32). Through the MAPK-ERK pathway, BCMA and TACI can prevent cell apoptosis and reduce the production of the pro-apoptotic protein Bim (33), leading to an increase in plasma cells. By activating ERK, p38, and JNK signaling pathways, along with the NF-κB classical pathway and the AKT/FOXO1 pathway, APRIL bound to HSPG receptors expressed on plasma cells, effectively transmitting signals that promote plasma cell longevity and survival (16, 17, 34). Additionally, increased BCMA expression, induced by gamma-secretase inhibitors, can also enhance plasma cell longevity and plasma blast survival (35, 36). And the high expression of TACI (37) on plasma cells may promote the expression of Blimp-1, which a key transcription factor involved in plasma cell development (38). Ultimately, these multiple pathways converged to facilitate the accumulation of plasma cells in the lacrimal gland lesions of IgG4-ROD patients. However, the mechanism by which APRIL promotes plasma cell aggregation in IgG4-ROD has not been fully elucidated and needs further exploration in the future.

Furthermore, we found a significant amount of secreted APRIL in lacrimal gland lesions of MALT lymphoma patients, consistent with the findings of Blosse et al. (22). Additionally, we discovered that APRIL acted on CD20^+^ B lymphocytes. These results suggested that APRIL could play a role in the regulation and development of B lymphocytes, with its overexpression potentially contributing to tumor-like proliferation of these cells. The specific mechanisms may involve APRIL binding to the BCMA receptor on the surface of B lymphocytes, which promoted their survival, maturation, and differentiation (39, 40). Furthermore, APRIL stimulated the NF-κB classical pathway (41) and maintains the expression of Blimp-1 (42) through the TACI receptor on B lymphocytes, facilitating their differentiation into long-lived antibody-secreting cells. This ultimately leads to tumor-like proliferation of B lymphocytes within the lacrimal gland lesions of MALT lymphoma patients. However, the mechanism by which APRIL promotes B-lymphocyte tumor-like proliferation in MALT lymphomas has not been fully elucidated and needs further exploration in the future.

In lacrimal gland lesions of MALT lymphoma patients with elevated IgG4, we observed significantly increased expression levels of CD20, CD138, and secreted APRIL. Through triple immunofluorescence staining, we found that APRIL acted on both CD20^+^ B lymphocytes and CD138^+^ plasma cells, leading to their proliferation. APRIL may have dual effects in these lesions, promoting both plasma cell proliferation and IgG4 production, as well as stimulating B lymphocyte proliferation.

In lacrimal gland lesions, IgG4-ROD could progress to MALT lymphoma, aligning with previous findings (23, 24). However, the mechanism of transformation from IgG4-ROD to MALT lymphoma needs further investigation.

This study also analyzed the relationship between CD138 levels, APRIL expression, and serum IgG4 levels in lacrimal gland lesions of IgG4-ROD patients. For the first time, we found a positive correlation between the expression level and positive rate of secreted APRIL and the serum IgG4 level in these lesions. Specifically, the positive rate and the colocalization of CD138 and APRIL were also positively correlated with serum IgG4 levels. This suggests that APRIL may enhance serum IgG4 secretion by acting on CD138^+^ plasma cells. After APRIL bound to HSPG, it activated two distinct signaling pathways via the TACI and BCMA receptors, which upregulated class switching of immunoglobulins and promoted the conversion of other immunoglobulin types (such as IgM and IgA) to IgG4 (17). Additionally, APRIL can prolong the lifespan of plasma cells (17, 18), leading to their aggregation and increased secretion of IgG4 upon interaction with CD138^+^ plasma cells, thereby elevating IgG4 levels.

This study has some limitations. First, the sample size of this study is small, and it is necessary to expand the sample size in the future to study the role of APRIL in IgG4-ROD and MALT lymphoma lacrimal gland lesions. Another limitation is that we have not yet verified the role of APRIL in IgG4-ROD and MAIL lymphoma. Therefore, further validation at both the cellular and animal levels is needed to comprehensively elucidate the role of APRIL in IgG4-ROD and MALT lymphoma lacrimal gland lesions and to identify new therapeutic targets for them.

In conclusion, APRIL is highly expressed in lacrimal gland lesions of IgG4-ROD patients, where it may act on CD138^+^ plasma cells to promote their enrichment and may also be associated with increased serum IgG4 levels. Additionally, APRIL is also highly expressed in lacrimal gland lesions of MALT lymphoma patients, potentially acting on CD20^+^ B lymphocytes to stimulate their proliferation. Ultimately, APRIL may play a role in the potential transformation of IgG4-ROD into MALT lymphoma.

## Data Availability

The original contributions presented in the study are included in the article/**Supplementary Material**. Further inquiries can be directed to the corresponding author.

## References

[1] Wallace ZS Perugino C Matza M Deshpande V Sharma A Stone JH. Immunoglobulin G4-related disease. Clin Chest Med. (2019) 40:583–97. doi: 10.1016/j.ccm.2019.05.005 PMC7133392 31376893

[2] Zen Y Fujii T Harada K Kawano M Yamada K Takahira M. Th2 and regulatory immune reactions are increased in immunoglobin G4-related sclerosing pancreatitis and cholangitis. Hepatology. (2007) 45:1538–46. doi: 10.1002/hep.21697 17518371

[3] Akiyama M Yasuoka H Yamaoka K Suzuki K Kaneko Y Kondo H. Enhanced IgG4 production by follicular helper 2 T cells and the involvement of follicular helper 1 T cells in the pathogenesis of IgG4-related disease. Arthritis Res Ther. (2016) 18:167. doi: 10.1186/s13075-016-1064-4 27411315 PMC4944254

[4] Mattoo H Mahajan VS Maehara T Deshpande V Della-Torre E Wallace ZS. Clonal expansion of CD4(+) cytotoxic T Lymphocytes in patients with IgG4-related disease. J Allergy Clin Immunol. (2016) 138:825–38. doi: 10.1016/j.jaci.2015.12.1330 PMC5014627 26971690

[5] Mattoo H Mahajan VS Della-Torre E Sekigami Y Carruthers M Wallace ZS. *De novo* oligoclonal expansions of circulating plasmablasts in active and relapsing IgG4-related disease. J Allergy Clin Immunol. (2014) 134:679–87. doi: 10.1016/j.jaci.2014.03.034 PMC4149918 24815737

[6] Della-Torre E Feeney E Deshpande V Mattoo H Mahajan V Kulikova M. B-cell depletion attenuates serological biomarkers of fibrosis and myofibroblast activation in IgG4-related disease. Ann Rheum Dis. (2015) 74:2236–43. doi: 10.1136/annrheumdis-2014-205799 PMC4806785 25143523

[7] Furukawa S Moriyama M Tanaka A Maehara T Tsuboi H Iizuka M. Preferential M2 macrophages contribute to fibrosis in IgG4-related dacryoadenitis and sialoadenitis, so-called Mikulicz’s disease. Clin Immunol. (2015) 156:9–18. doi: 10.1016/j.clim.2014.10.008 25450336

[8] Stone JH Zen Y Deshpande V. IgG4-related disease. N Engl J Med. (2012) 366:539–51. doi: 10.1056/NEJMra1104650 22316447

[9] Takeuchi M Sato Y Ohno K Tanaka S Takata K Gion Y. T helper 2 and regulatory T-cell cytokine production by mast cells: a key factor in the pathogenesis of IgG4-related disease. Mod Pathol. (2014) 27:1126–36. doi: 10.1038/modpathol.2013.236 24390219

[10] Della-Torre E Lanzillotta M Doglioni C. Immunology of igg4-related disease. Clin Exp Immunol. (2015) 181:191–206. doi: 10.1111/cei.12641 25865251 PMC4516435

[11] Deshpande V Zen Y Chan JK Yi EE Sato Y Yoshino T. Consensus statement on the pathology of IgG4 related disease. Mod Pathol. (2012) 25:1181–92. doi: 10.1038/modpathol.2012.72 22596100

[12] Sato Y Notohara K Kojima M Takata K Masaki Y Yoshino T. IgG4-related disease: historical overview and pathology of hematological disorders. Pathol Int. (2010) 60:247–58. doi: 10.1111/j.1440-1827.2010.02524.x 20403026

[13] Min HK Lee YS Yang SW Lee J Kwok SK Ju JH. Clinical outcomes and pathological characteristics of immunoglobulin G4-related ophthalmic disease versus orbital inflammatory pseudotumor. Korean J Intern Med. (2019) 34:220–6. doi: 10.3904/kjim.2016.304 PMC6325444 29050463

[14] Huard B McKee T Bosshard C Durual S Matthes T Myit S. APRIL secreted by neutrophils binds to heparan sulfate proteoglycans to create plasma cell niches in human mucosa. J Clin Invest. (2008) 18:2887–95. doi: 10.1172/JCI33760 PMC2447926 18618015

[15] Stein JV López-Fraga M Elustondo FA Carvalho-Pinto CE Rodríguez D Gómez-Caro R. APRIL modulates B and T cell immunity. J Clin Invest. (2002) 109:1587–98. doi: 10.1172/JCI15034 PMC151011 12070306

[16] Mhawech-Fauceglia P Kaya G Sauter G McKee T Donze O Schwaller J. The source of APRIL up-regulation in human solid tumor lesions. J Leukoc Biol. (2006) 80:697–704. doi: 10.1189/jlb.1105655 16793914

[17] Vincent FB Morand EF Schneider P Mackay F. The BAFF/APRIL system in SLE pathogenesis. Nat Rev Rheumatol. (2014) 10:365–73. doi: 10.1038/nrrheum.2014.33 24614588

[18] Yu G Boone T Delaney J Hawkins N Kelley M Ramakrishnan M. APRIL and TALL-I and receptors BCMA and TACI: system for regulating humoral immunity. Nat Immunol. (2000) 1:252–6. doi: 10.1038/79802 10973284

[19] Ingold K Zumsteg A Tardivel A Huard B Steiner QG Cachero TG. Identification of proteoglycans as the APRIL specific binding partners. J Exp Med. (2005) 201:1375–83. doi: 10.1084/jem.20042309 PMC2213192 15851487

[20] Hendriks J Planelles L de-Jong-Odding J Hardenberg G Pals ST Hahne M. Heparan sulfate proteoglycan binding promotes APRIL-induced tumor cell proliferation. Cell Death Differ. (2005) 12:637–48. doi: 10.1038/sj.cdd.4401647 15846369

[21] Kawakami T Mizushima I Yamada K Fujii H Ito K Yasuno T. Abundant a proliferation-inducing ligand (APRIL)-producing macrophages contribute to plasma cell accumulation in immunoglobulin G4-related disease. Nephrol Dial Transpl. (2019) 34:960–9. doi: 10.1093/ndt/gfy296 PMC6545467 30325430

[22] Blosse A Peru S Levy M Marteyn B Floch P Sifré E. APRIL−producing eosinophils are involved in gastric MALT lymphomagenesis induced by Helicobacter sp infection. Sci Rep. (2020) 10:14858. doi: 10.1038/s41598-020-71792-3 32908188 PMC7481773

[23] Cheuk W Yuen HK Chan AC Shih LY Kuo TT Ma MW. Ocular adnexal lymphoma associated with IgG4+ chronic sclerosing dacryoadenitis: a previously undescribed complication of IgG4-related sclerosing disease. Am J Surg Pathol. (2008) 32:1159–67. doi: 10.1097/PAS.0b013e31816148ad 18580683

[24] Carruthers MN Khosroshahi A Augustin T Deshpande V Stone JH. The diagnostic utility of serum IgG4 concentrations in IgG4-related disease. Ann Rheum Dis. (2015) 74:14–8. doi: 10.1136/annrheumdis-2013-204907 24651618

[25] Goto H Takahira M Azumi A. Diagnostic criteria for IgG4-related ophthalmic disease. Jpn J Ophthalmol. (2015) 59:1–7. doi: 10.1007/s10384-014-0352-2 25392273

[26] Kusuda T Uchida K Miyoshi H Koyabu M Satoi S Takaoka M. Involvement of inducible costimulatory- and interleukin 10-positive regulatory T cells in the development of IgG4-related autoimmune pancreatitis. Pancreas. (2011) 40:1120–30. doi: 10.1097/MPA.0b013e31821fc796 21926547

[27] Takeuchi M Ohno K Takata K Gion Y Tachibana T Orita Y. Interleukin 13-positive mast cells are increased in immunoglobulin G4-related sialadenitis. Sci Rep. (2015) 5:7696. doi: 10.1038/srep07696 25571893 PMC4287729

[28] Litinskiy MB Nardelli B Hilbert DM He B Schaffer A Casali P. DCs induce CD40-independent immunoglobulin class switching through BLyS and APRIL. Nat Immunol. (2002) 3:822–9. doi: 10.1038/ni829 PMC4621779 12154359

[29] Schwaller J Schneider P Mhawech-Fauceglia P McKee T Myit S Matthes T. Neutrophil-derived APRIL concentrated in tumor lesions by proteoglycans correlates with human B-cell lymphoma aggressiveness. Blood. (2007) 109:331–8. doi: 10.1182/blood-2006-02-001800 17190854

[30] Hatzoglou A Roussel J Bourgeade MF Rogier E Madry C Inoue J. TNF receptor family member BCMA (B cell maturation) associates with TNF receptor-associated factor (TRAF) 1, TRAF2, and TRAF3 and activates NF-kappa B, elk-1, c-Jun N-terminal kinase, and p38 mitogen-activated protein kinase. J Immunol. (2000) 165:1322–30. doi: 10.4049/jimmunol.165.3.1322 10903733

[31] Bossen C Schneider P. BAFF, APRIL and their receptors: structure, function and signaling. Semin Immunol. (2006) 18:263–75. doi: 10.1016/j.smim.2006.04.006 16914324

[32] Mackay F Schneider P Rennert P Browning J. BAFF and APRIL: a tutorial on B cell survival. Annu Rev Immunol. (2003) 21:231–64. doi: 10.1146/annurev.immunol.21.120601.141152 12427767

[33] Craxton A Draves KE Gruppi A Clark EA. BAFF regulates B cell survival by down regulating the BH3-only family member bim via the ERK pathway. J Exp Med. (2005) 202:1363–74. doi: 10.1084/jem.20051283 PMC2212971 16301744

[34] Bret C Hose D Reme T Sprynski AC Mahtouk K Schved JF. Expression of genes encoding for proteins involved in heparan sulphate and chondroitin sulphate chain synthesis and modification in normal and Malignant plasma cells. Br J Haematol. (2009) 145:350–68. doi: 10.1111/j.1365-2141.2009.07633.x PMC2730414 19298595

[35] Laurent SA Hoffmann FS Kuhn PH Cheng Q Chu Y Schmidt-Supprian M. [amp]]gamma;-Secretase directly sheds the survival receptor BCMA from plasma cells. Nat Commun. (2015) 6:7333. doi: 10.1038/ncomms8333 26065893 PMC4490565

[36] Stephenson S Care MA Doody GM Tooze RM. APRIL drives a coordinated but diverse response as a foundation for plasma cell longevity. J Immunol. (2022) 209:926–37. doi: 10.4049/jimmunol.2100623 PMC7613700 36130130

[37] Ng LG Sutherland AP Newton R Qian F Cachero TG Scott ML. B cell-activating factor belonging to the TNF family (BAFF)-R is the principal BAFF receptor facilitating BAFF costimulation of circulating T and B cells. J Immunol. (2004) 173:807–17. doi: 10.4049/jimm-unol.173.2.807 15240667

[38] Tsuji S Cortesão C Bram RJ Platt JL Cascalho M. TACI deficiency impairs sustained Blimp-1 expression in B cells decreasing long-lived plasma cells in the bone marrow. Blood. (2011) 118:5832–9. doi: 10.1182/blood-2011-05-353961 PMC3228499 21984806

[39] Moore PA Belvedere O Orr A Pieri K LaFleur DW Feng P. BLyS: member of the tumor necrosis factor family and B lymphocyte stimulator. Science. (1999) 285:260–3. doi: 10.1126/science.285.5425.260 10398604

[40] Laabi Y Gras MP Brouet JC Berger R Larsen CJ Tsapis A. The BCMA gene, preferentially expressed during B lymphoid maturation, is bidirectionally transcribed. Nucleic Acids Res. (1994) 22:1147–54. doi: 10.1093/nar/22.7.1147 PMC523635 8165126

[41] Ozaki K Spolski R Ettinger R Kim HP Wang G Qi CF. Regulation of B cell differentiation and plasma cell generation by IL-21, a novel inducer of Blimp-1 and Bcl-6. J Immunol. (2004) 173:5361–71. doi: 10.4049/jimmunol.173.9.5361 15494482

[42] Xia XZ Treanor J Senaldi G Khare SD Boone T Kelley M. TACI is a TRAF-interacting receptor for TALL-1, a tumor necrosis factor family member involved in B cell regulation. J Exp Med. (2000) 192:137–43. doi: 10.1084/jem.192.1.137 PMC1887716 10880535

