# Supplementary figures and images for: Similarities and differences of a proliferation-inducing ligand expression in lacrimal gland lesions of patients with IgG4-associated ophthalmic diseases and mucosa-associated lymphoid tissue lymphoma
Source: Front Immunol. 2025 Feb 18;16:1514003. doi: 10.3389/fimmu.2025.1514003 (PMC11876129; doi:10.3389/fimmu.2025.1514003)

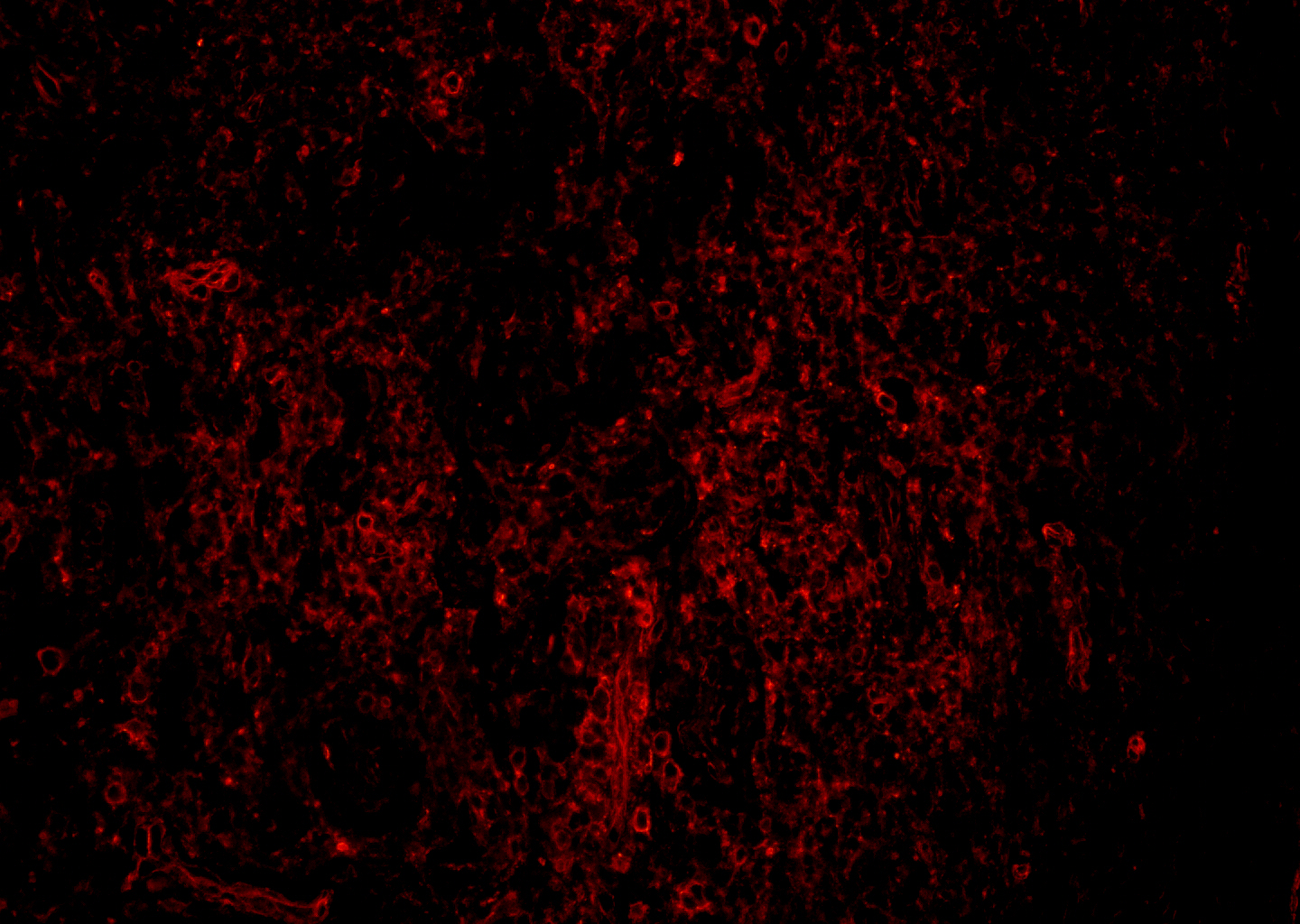

Supplement: Supplementary file 2 [file DataSheet1.zip › Supplementary Materials/IgG4-ROD/IgG4-7 CD138红+Aprily8绿 200-1.jpg]

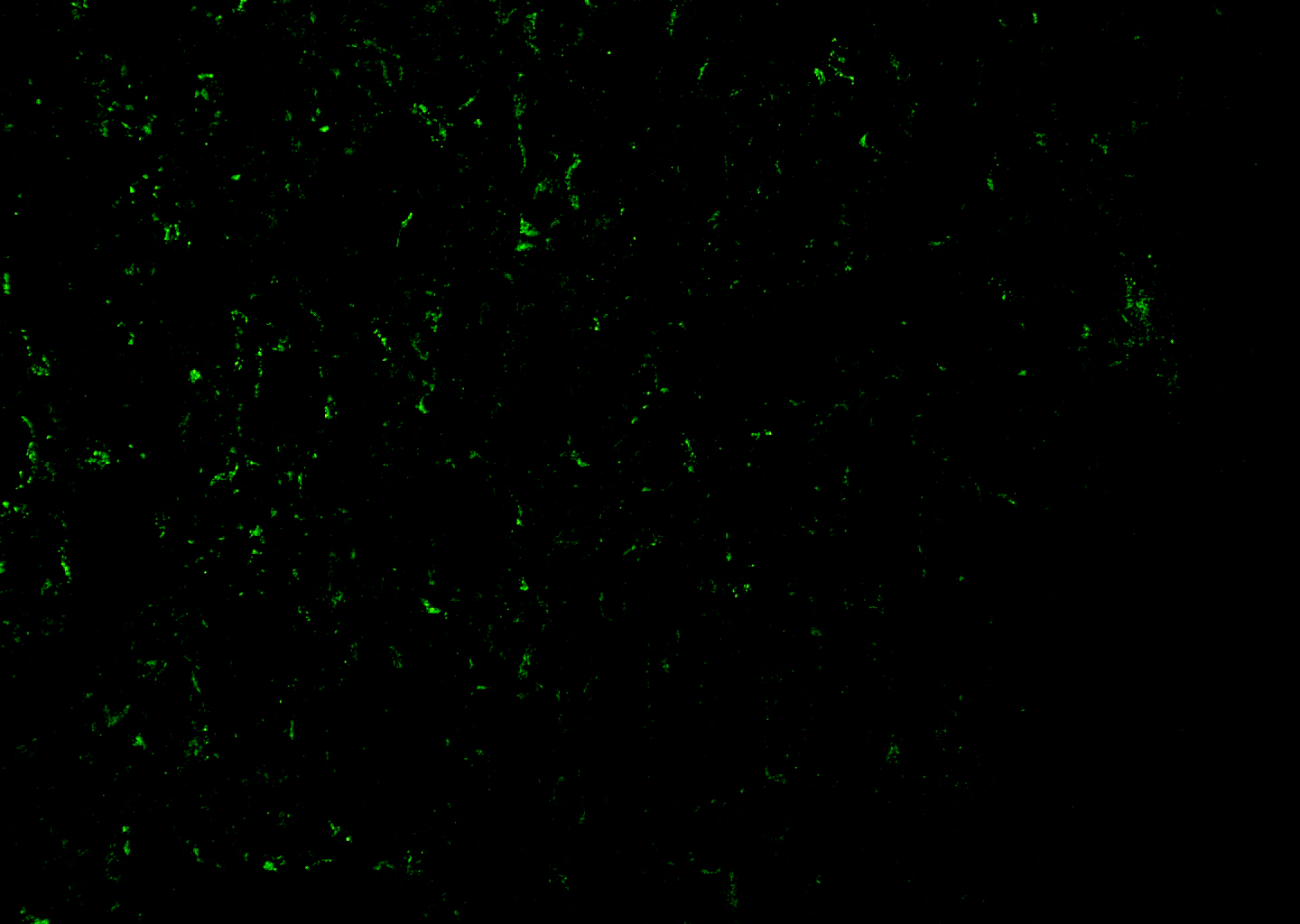

Supplement: Supplementary file 2 [file DataSheet1.zip › Supplementary Materials/IgG4-ROD/IgG4-7 CD138红+Aprily8绿 200-2.jpg]

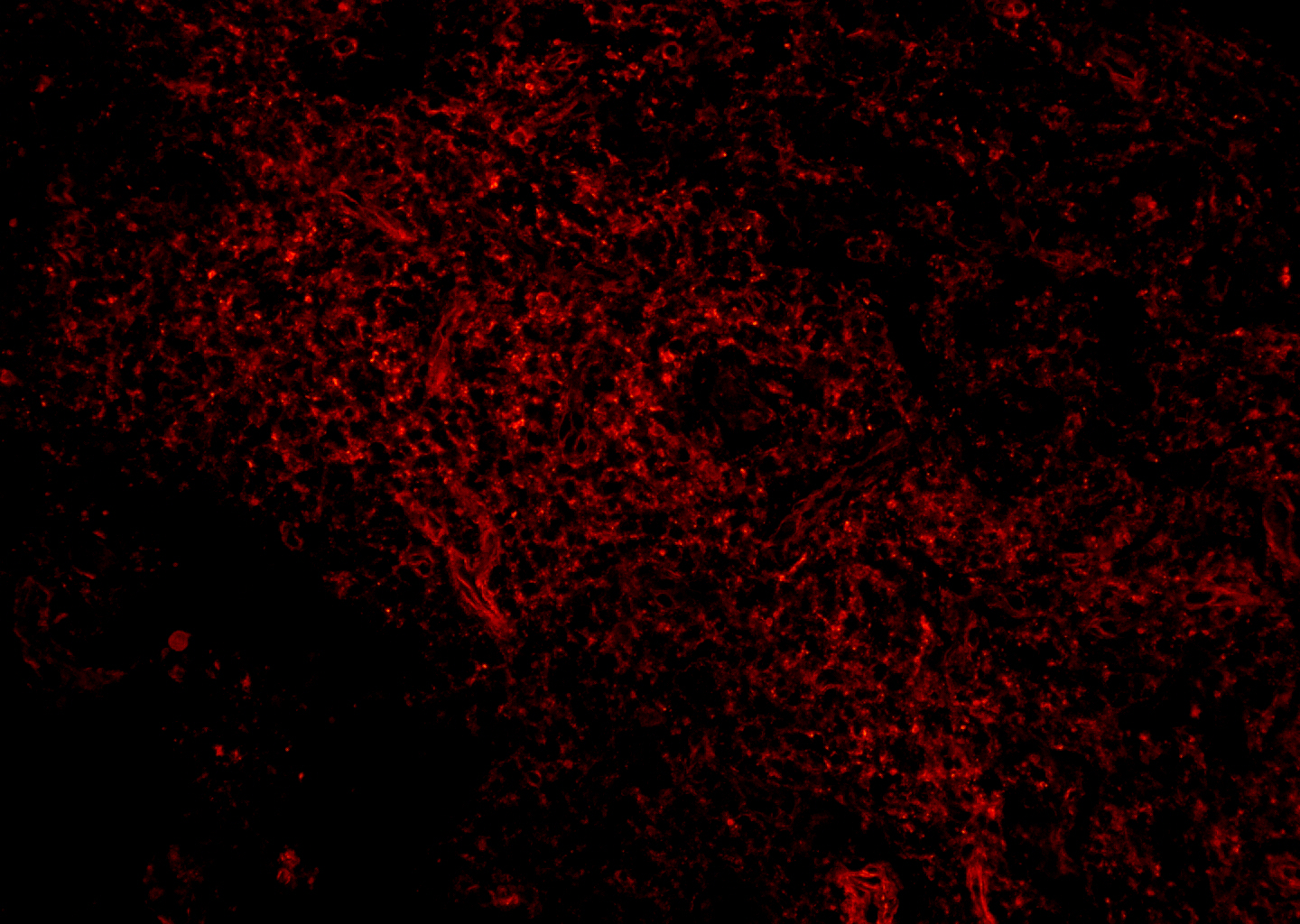

Supplement: Supplementary file 2 [file DataSheet1.zip › Supplementary Materials/IgG4-ROD/IgG4-7 CD138红+Aprily8绿 200-4.jpg]

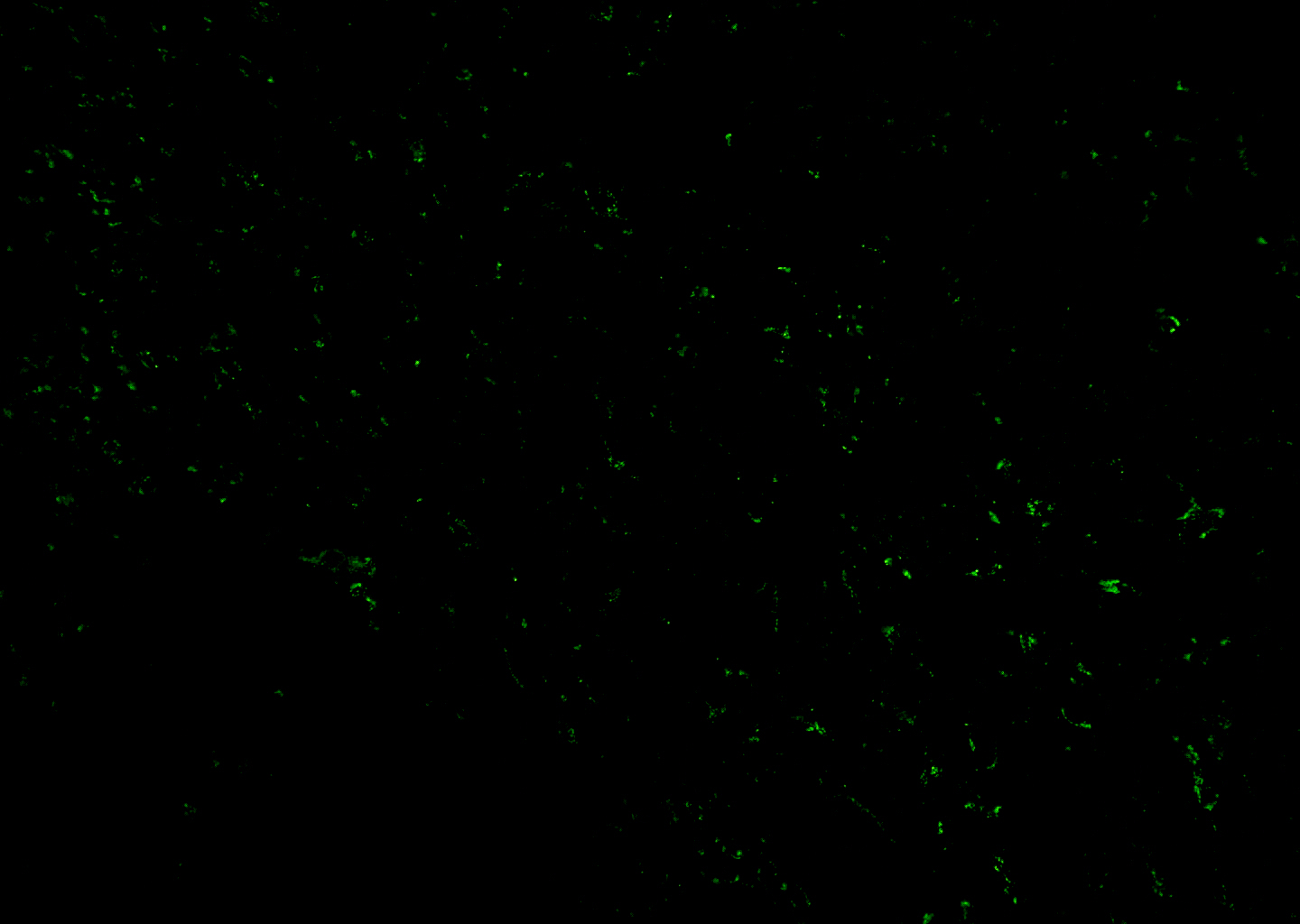

Supplement: Supplementary file 2 [file DataSheet1.zip › Supplementary Materials/IgG4-ROD/IgG4-7 CD138红+Aprily8绿 200-5.jpg]

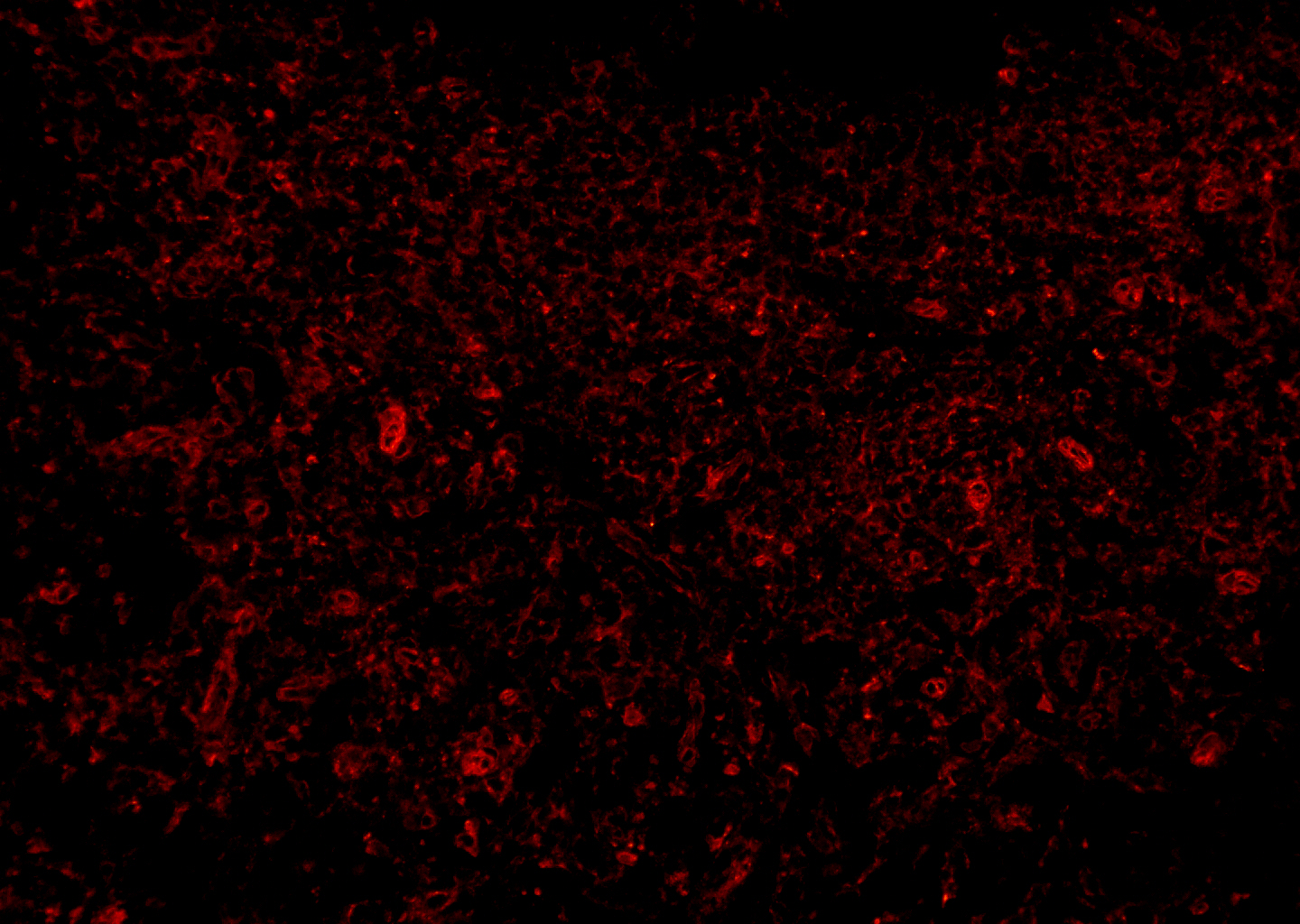

Supplement: Supplementary file 2 [file DataSheet1.zip › Supplementary Materials/IgG4-ROD/IgG4-7 CD138红+Aprily8绿 200-7.jpg]

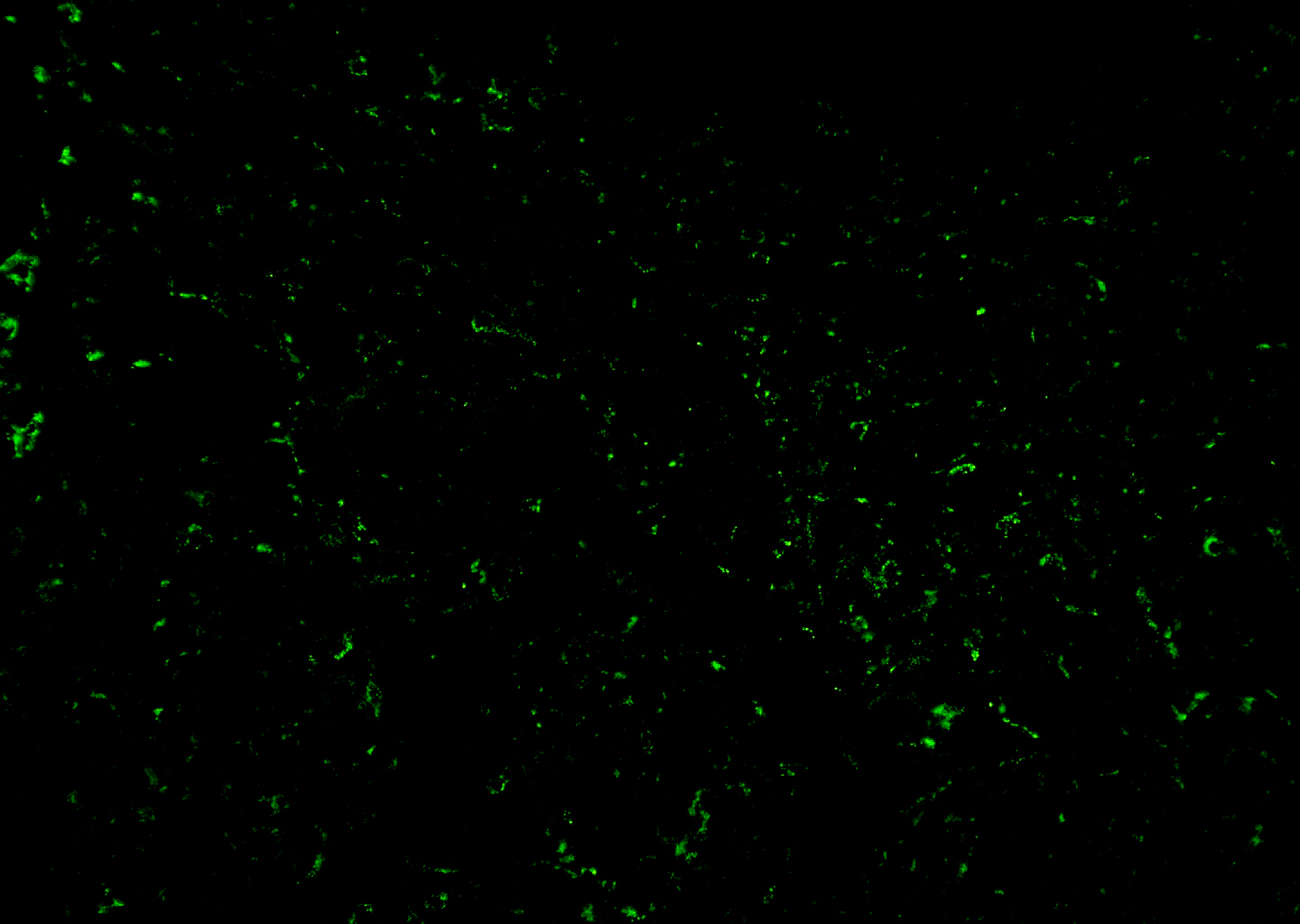

Supplement: Supplementary file 2 [file DataSheet1.zip › Supplementary Materials/IgG4-ROD/IgG4-7 CD138红+Aprily8绿 200-8.jpg]

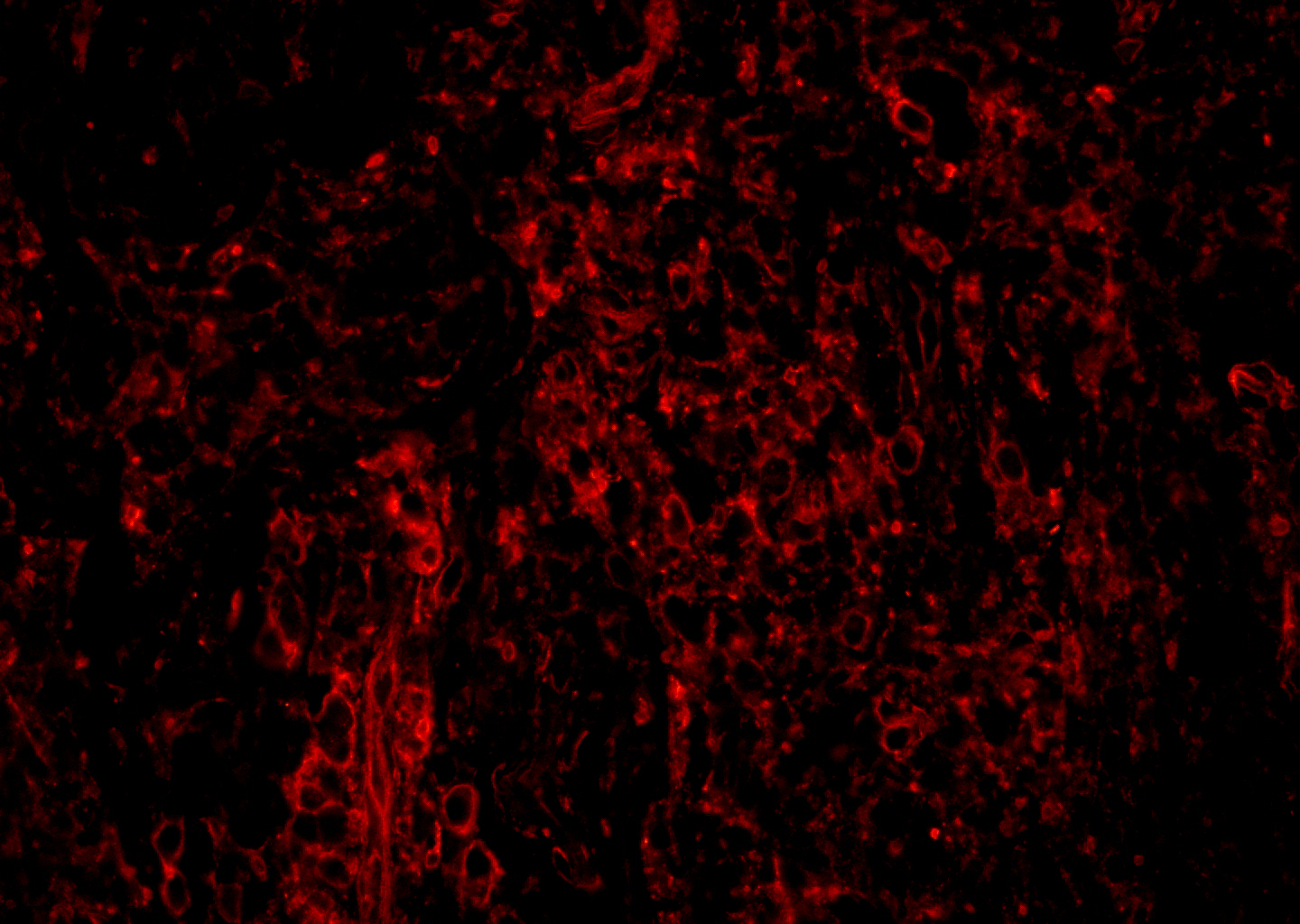

Supplement: Supplementary file 2 [file DataSheet1.zip › Supplementary Materials/IgG4-ROD/IgG4-7 CD138红+Aprily8绿 400-1.jpg]

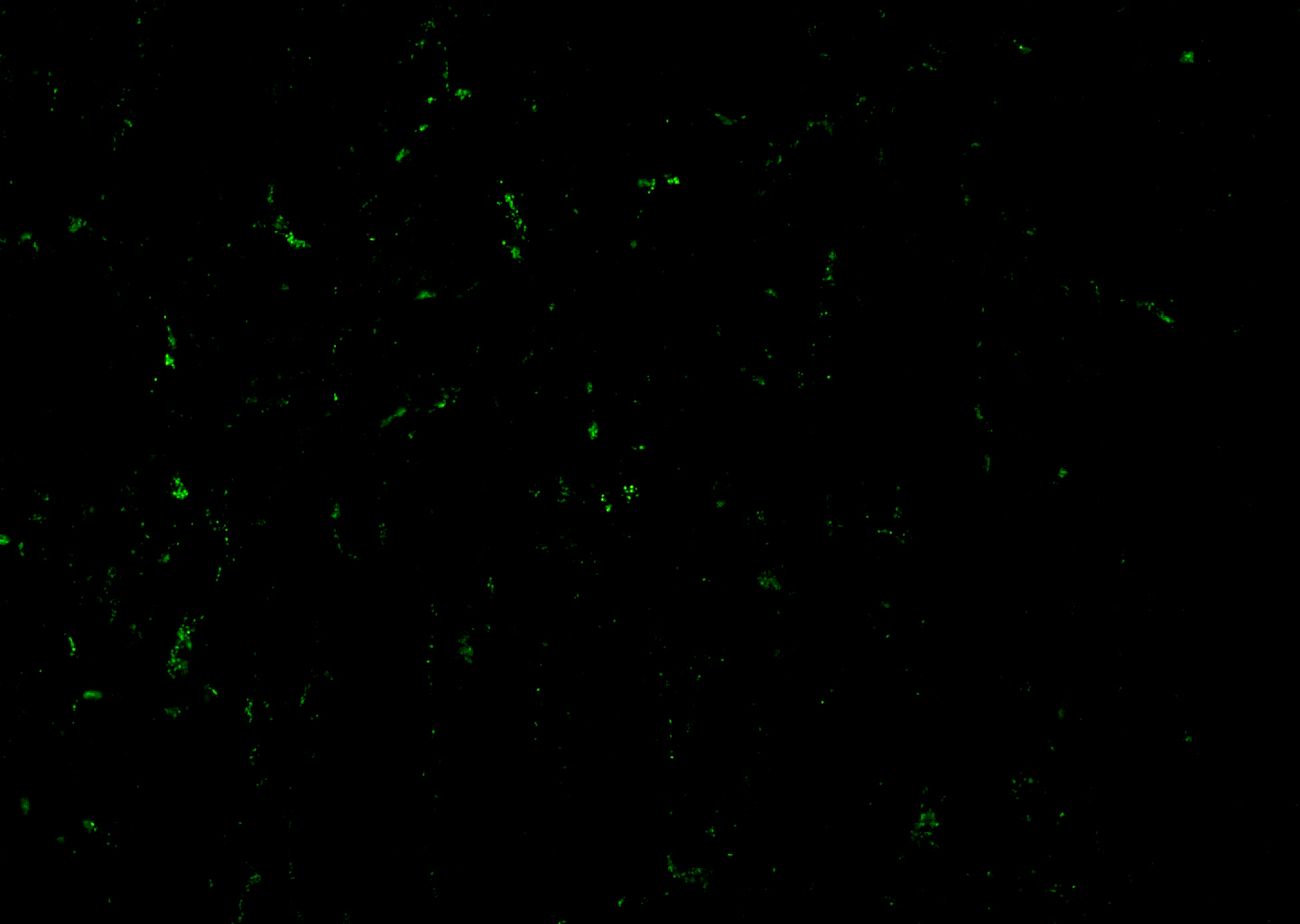

Supplement: Supplementary file 2 [file DataSheet1.zip › Supplementary Materials/IgG4-ROD/IgG4-7 CD138红+Aprily8绿 400-2.jpg]

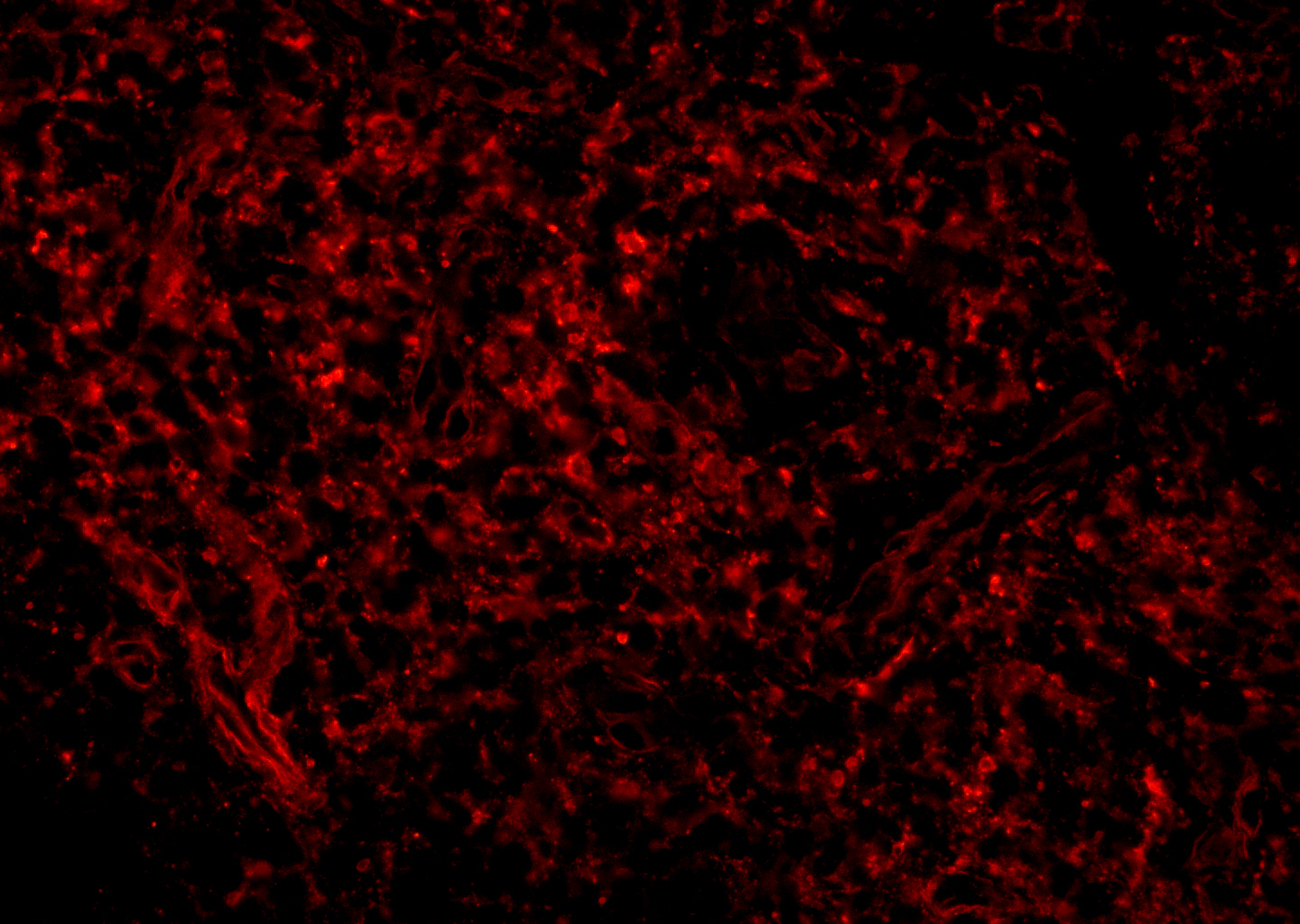

Supplement: Supplementary file 2 [file DataSheet1.zip › Supplementary Materials/IgG4-ROD/IgG4-7 CD138红+Aprily8绿 400-4.jpg]

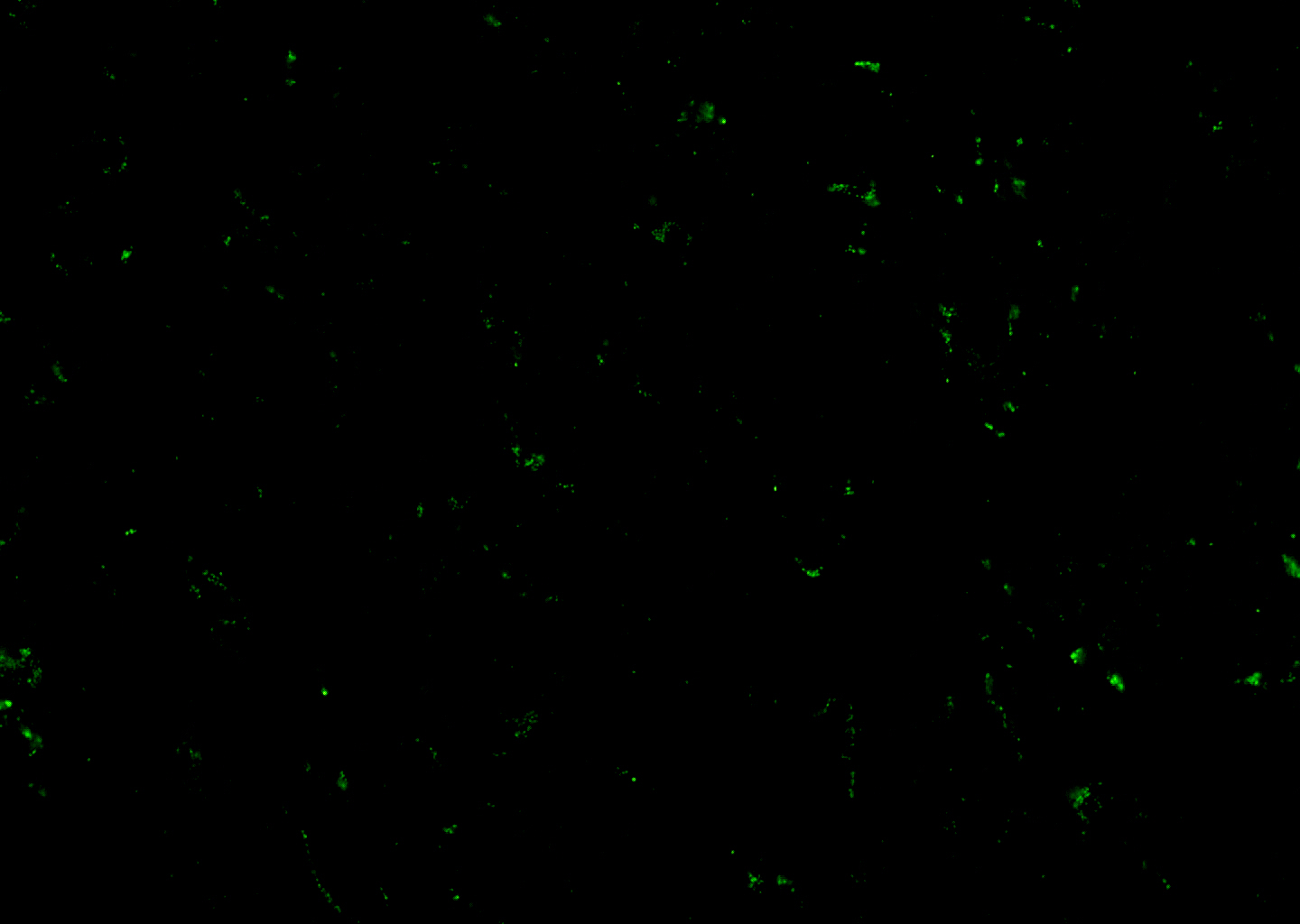

Supplement: Supplementary file 2 [file DataSheet1.zip › Supplementary Materials/IgG4-ROD/IgG4-7 CD138红+Aprily8绿 400-5.jpg]

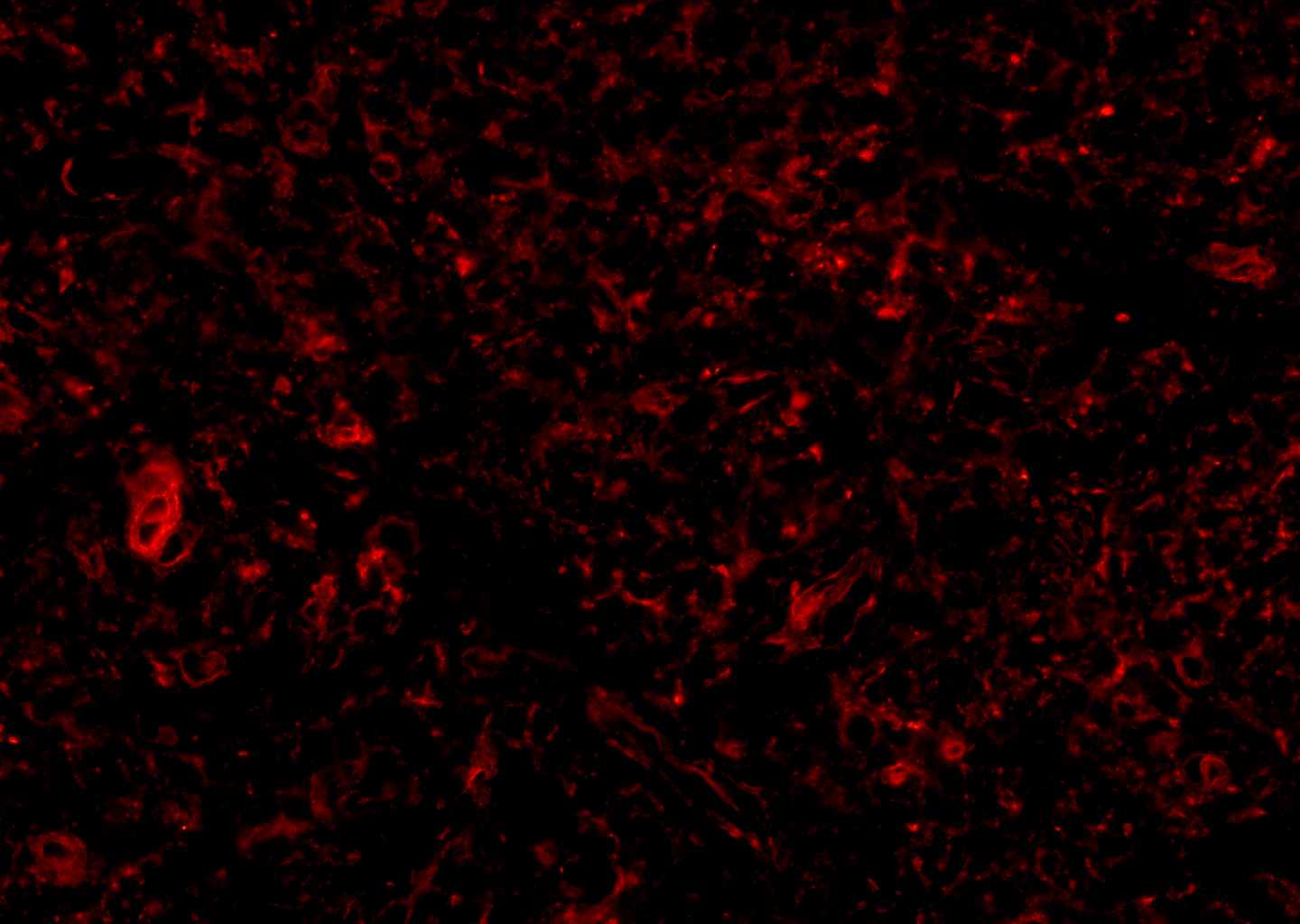

Supplement: Supplementary file 2 [file DataSheet1.zip › Supplementary Materials/IgG4-ROD/IgG4-7 CD138红+Aprily8绿 400-7.jpg]

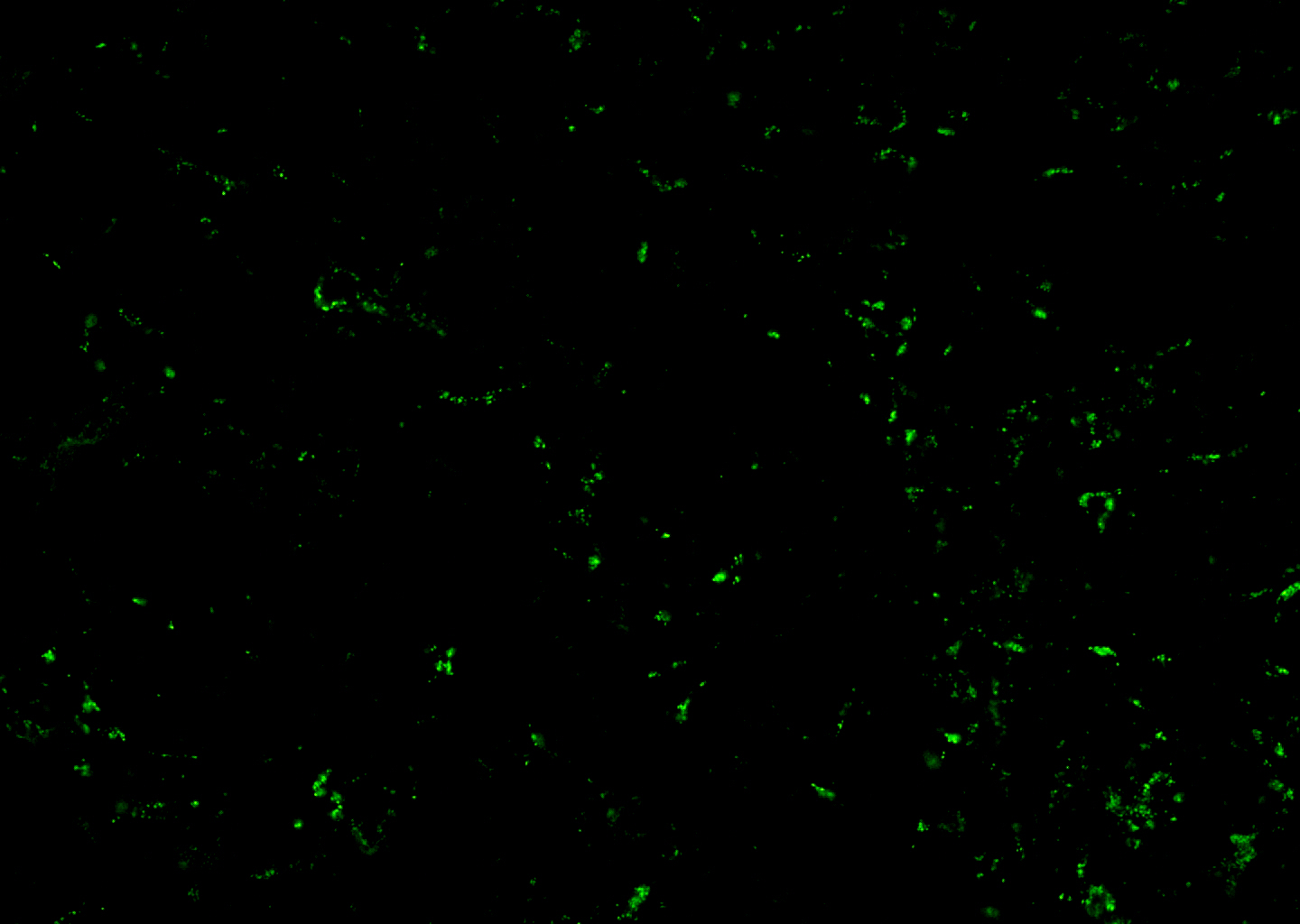

Supplement: Supplementary file 2 [file DataSheet1.zip › Supplementary Materials/IgG4-ROD/IgG4-7 CD138红+Aprily8绿 400-8.jpg]

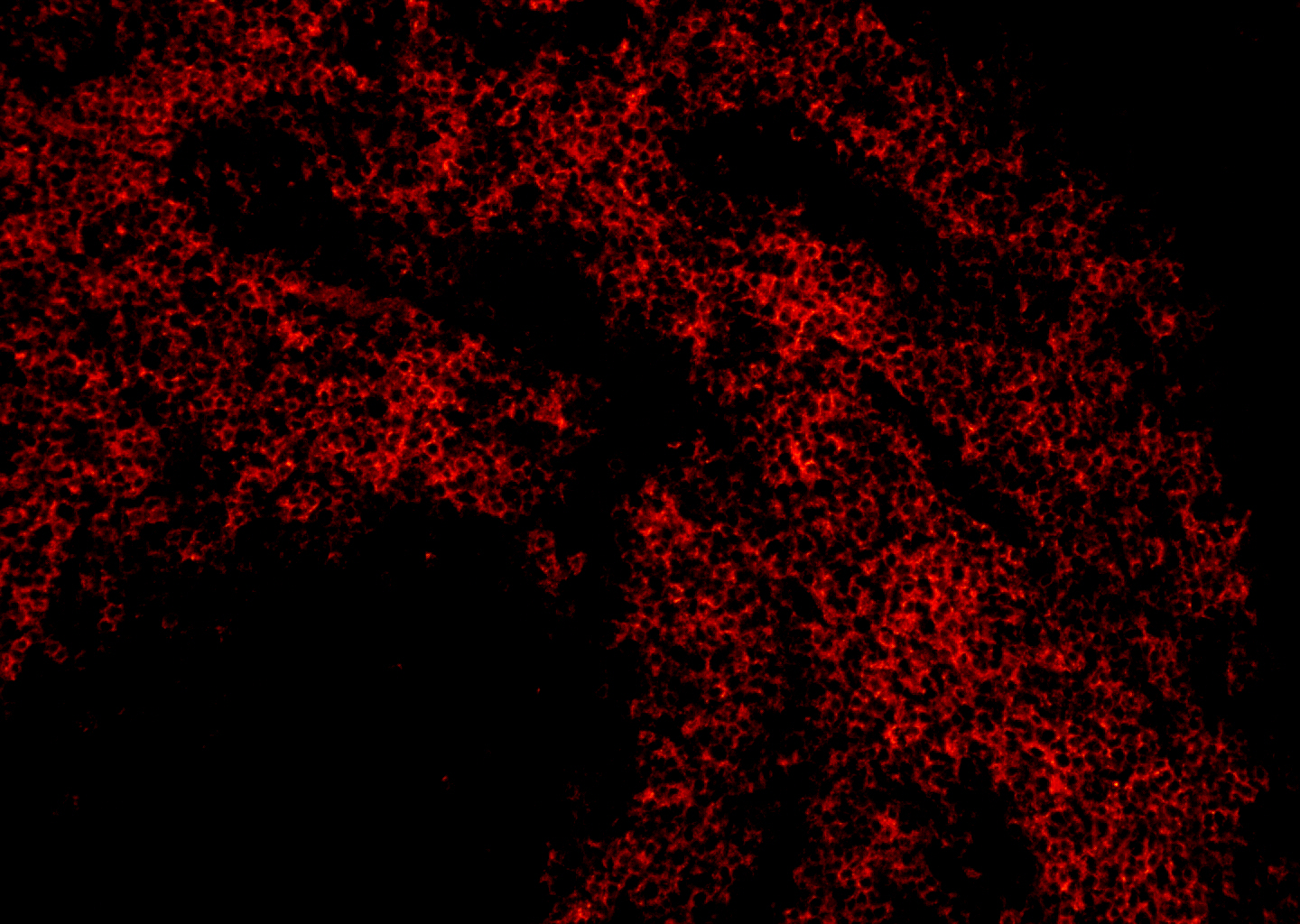

Supplement: Supplementary file 2 [file DataSheet1.zip › Supplementary Materials/MALT lymphoma/20号 CD20红+Aprily8绿 200-1.jpg]

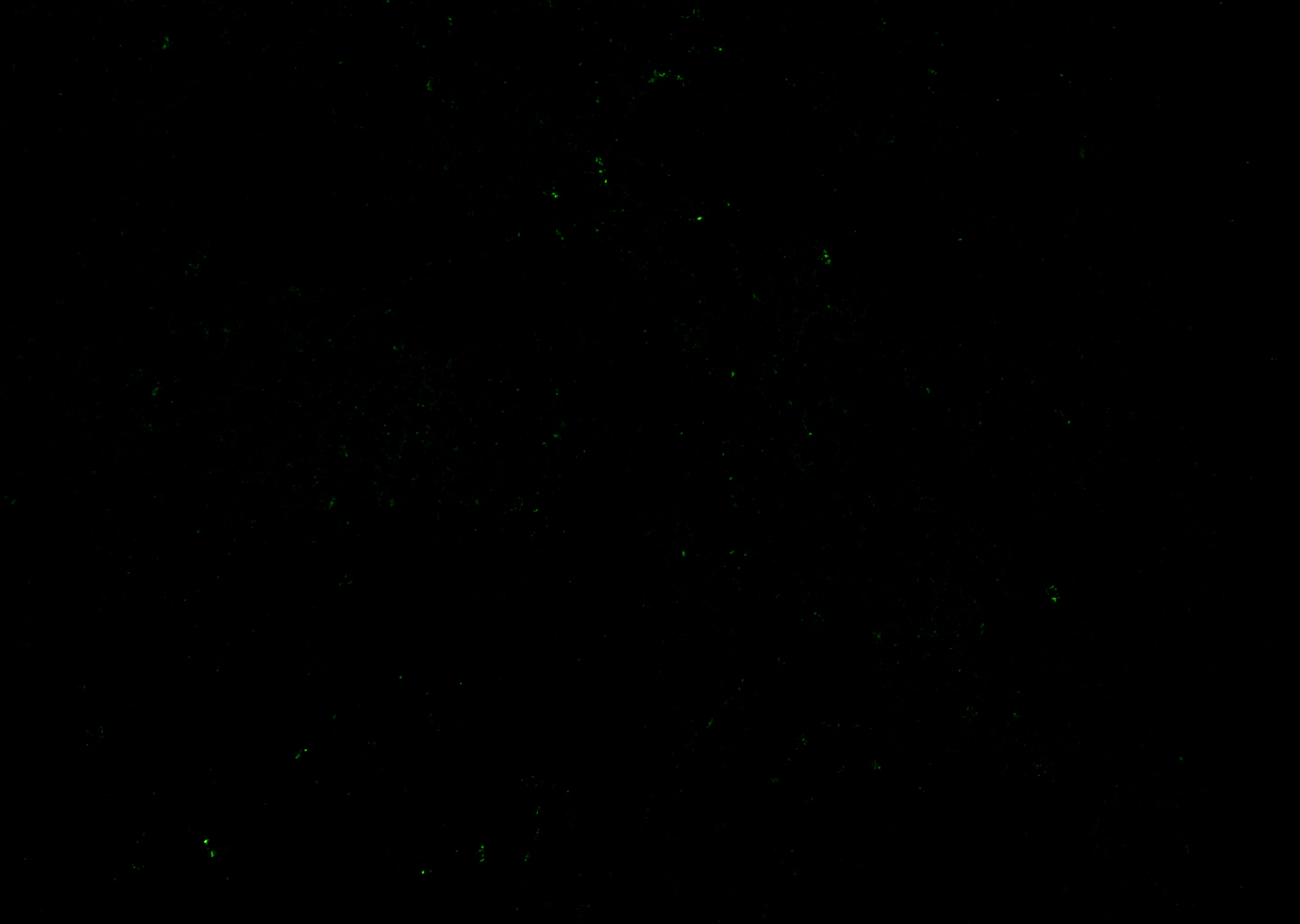

Supplement: Supplementary file 2 [file DataSheet1.zip › Supplementary Materials/MALT lymphoma/20号 CD20红+Aprily8绿 200-2.jpg]

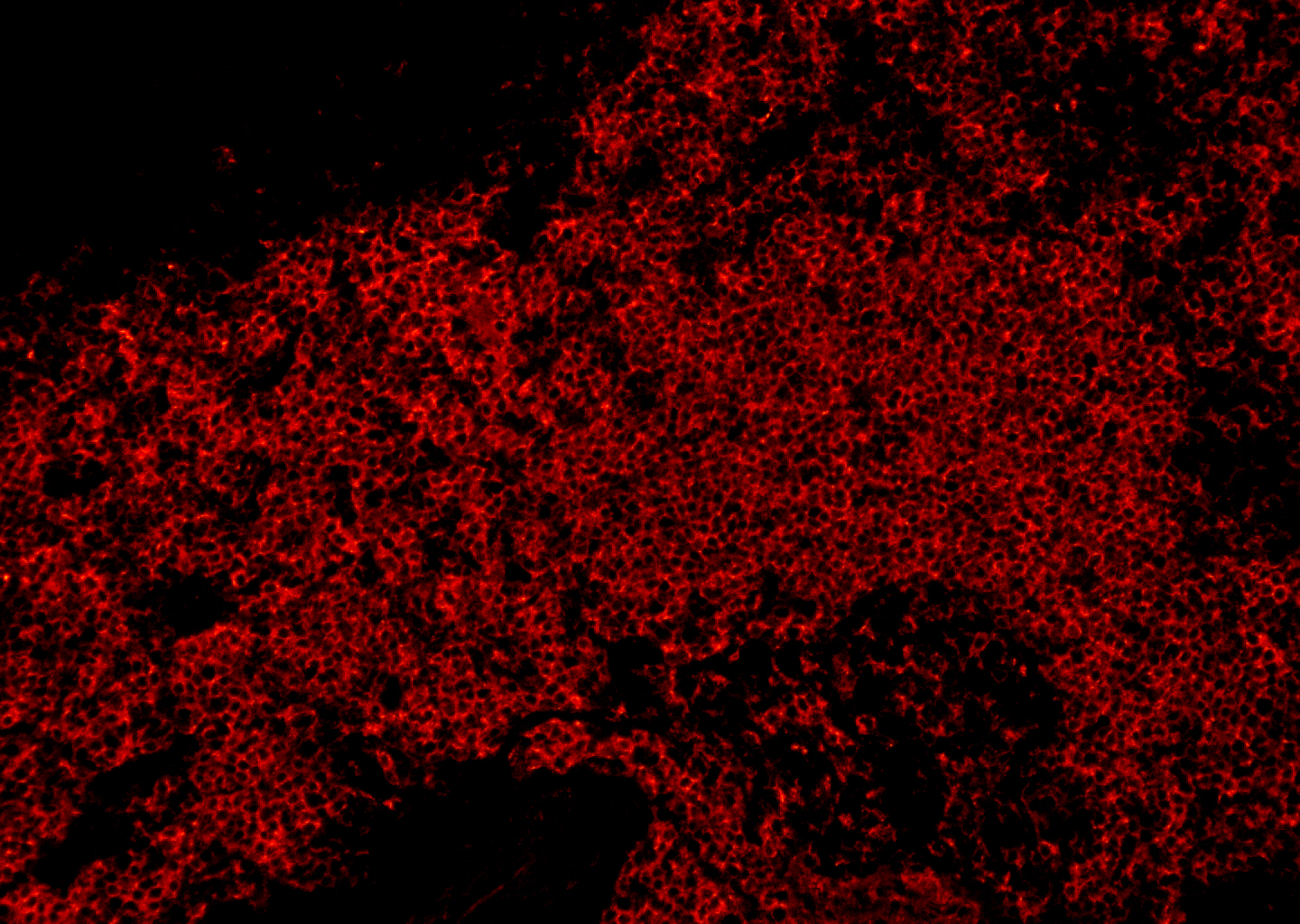

Supplement: Supplementary file 2 [file DataSheet1.zip › Supplementary Materials/MALT lymphoma/20号 CD20红+Aprily8绿 200-4.jpg]

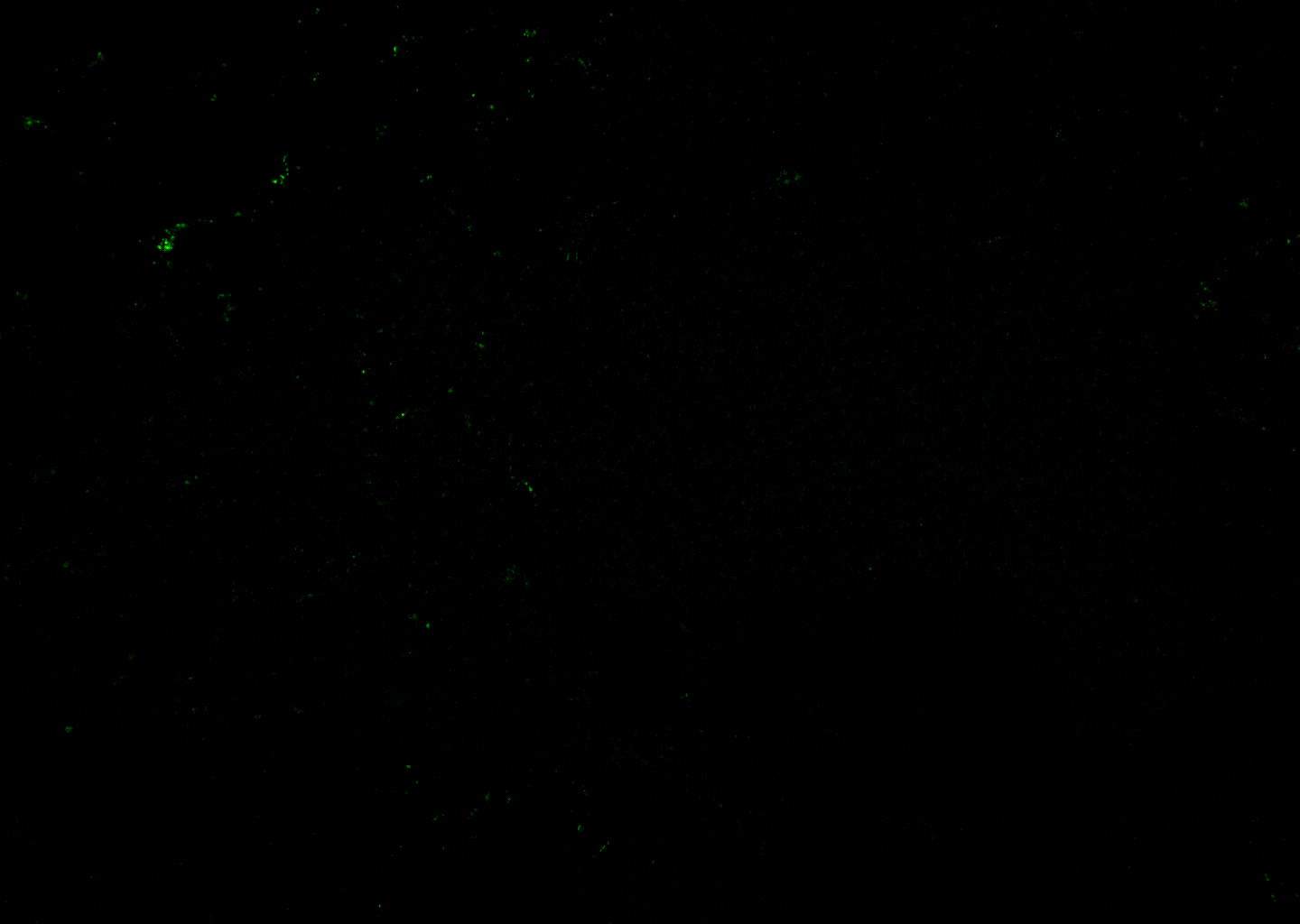

Supplement: Supplementary file 2 [file DataSheet1.zip › Supplementary Materials/MALT lymphoma/20号 CD20红+Aprily8绿 200-5.jpg]

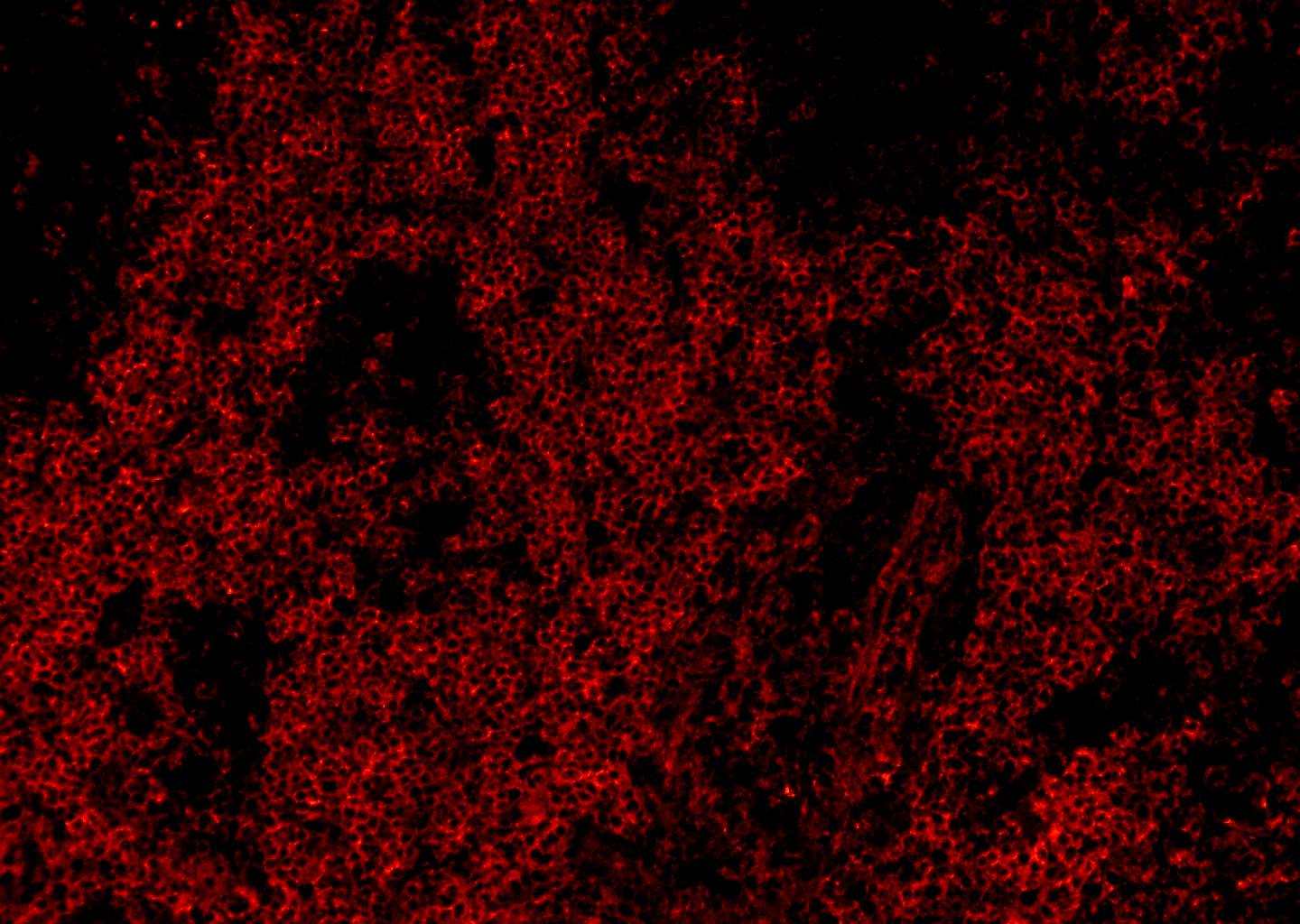

Supplement: Supplementary file 2 [file DataSheet1.zip › Supplementary Materials/MALT lymphoma/20号 CD20红+Aprily8绿 200-7.jpg]

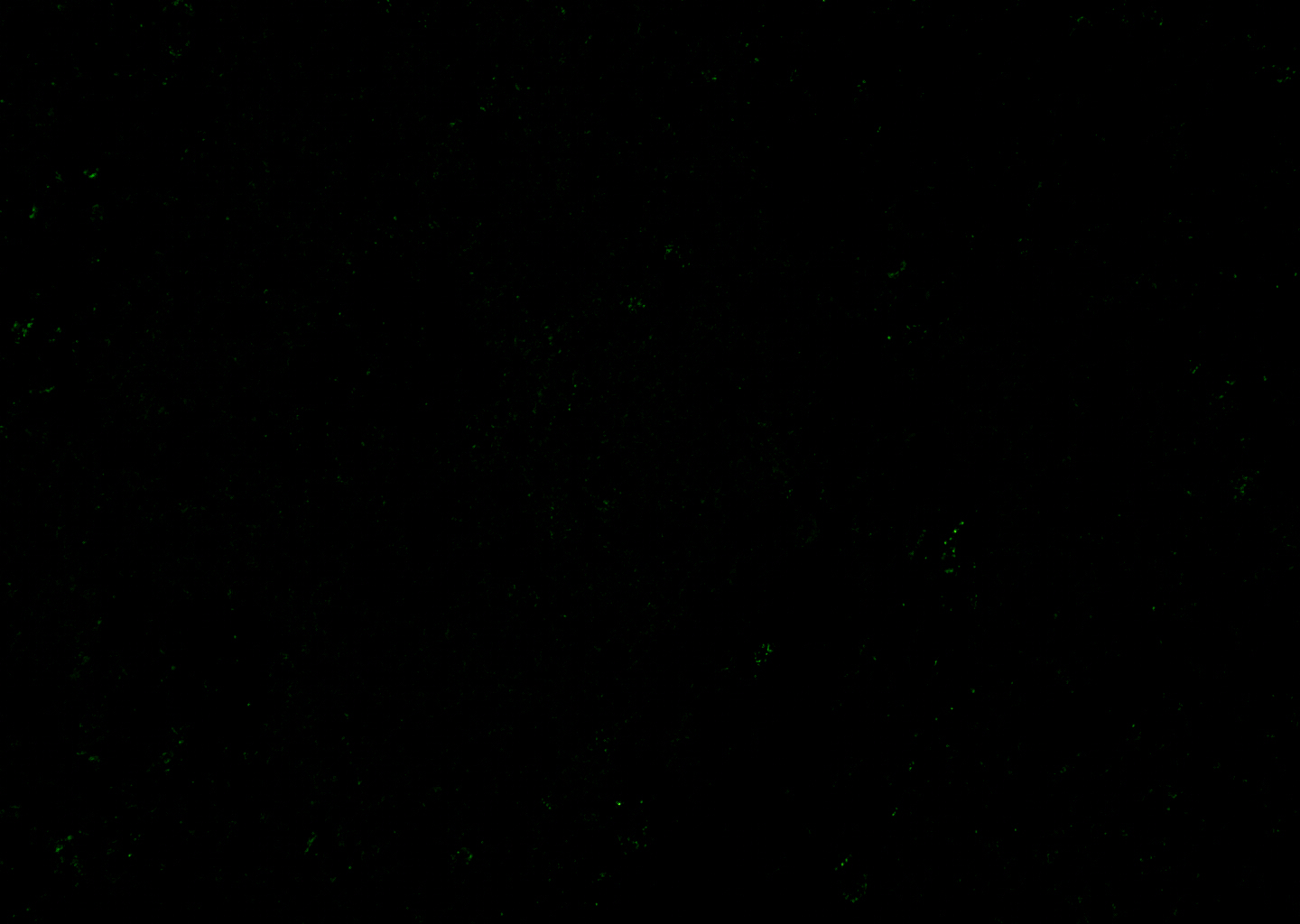

Supplement: Supplementary file 2 [file DataSheet1.zip › Supplementary Materials/MALT lymphoma/20号 CD20红+Aprily8绿 200-8.jpg]

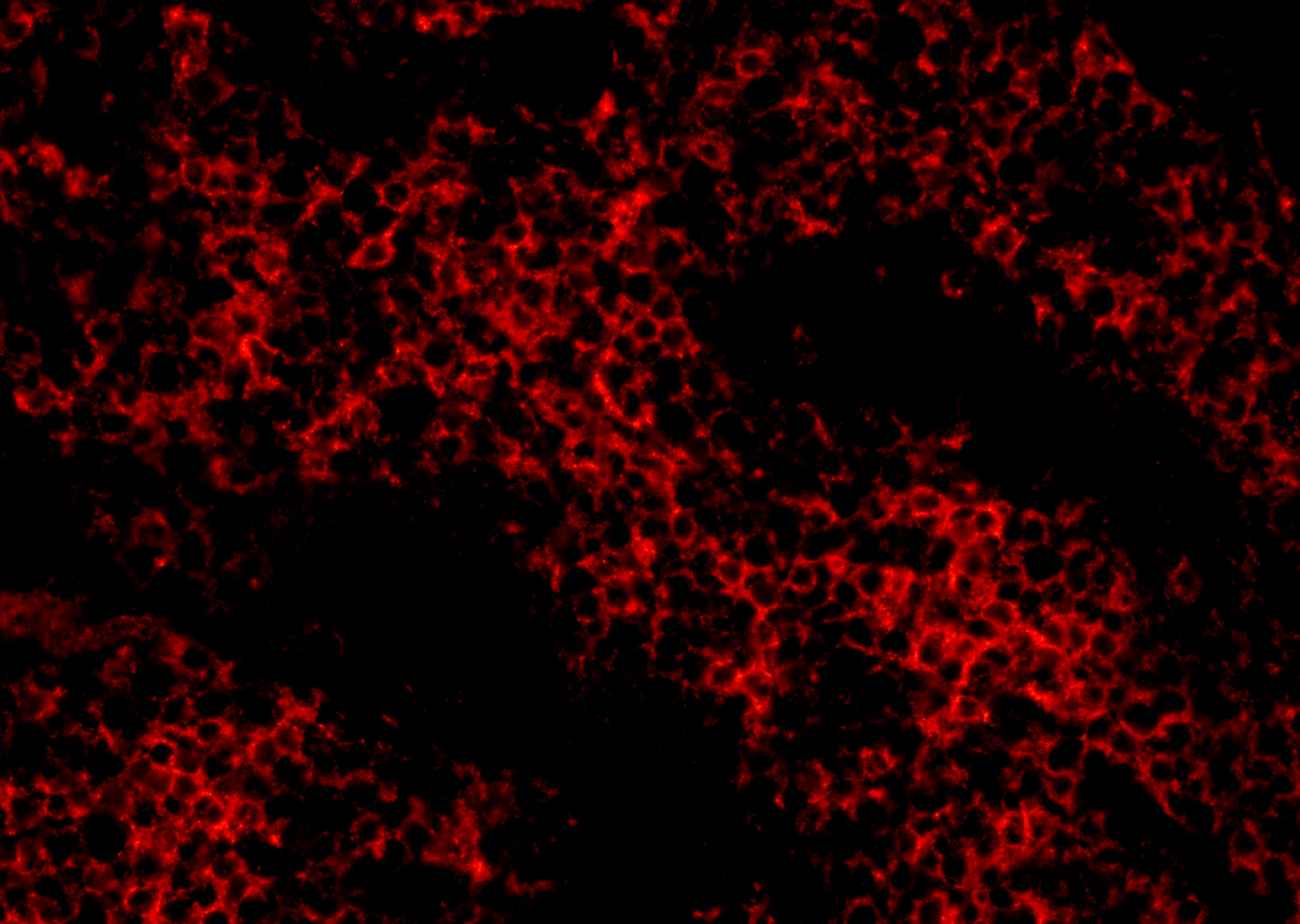

Supplement: Supplementary file 2 [file DataSheet1.zip › Supplementary Materials/MALT lymphoma/20号 CD20红+Aprily8绿 400-1.jpg]

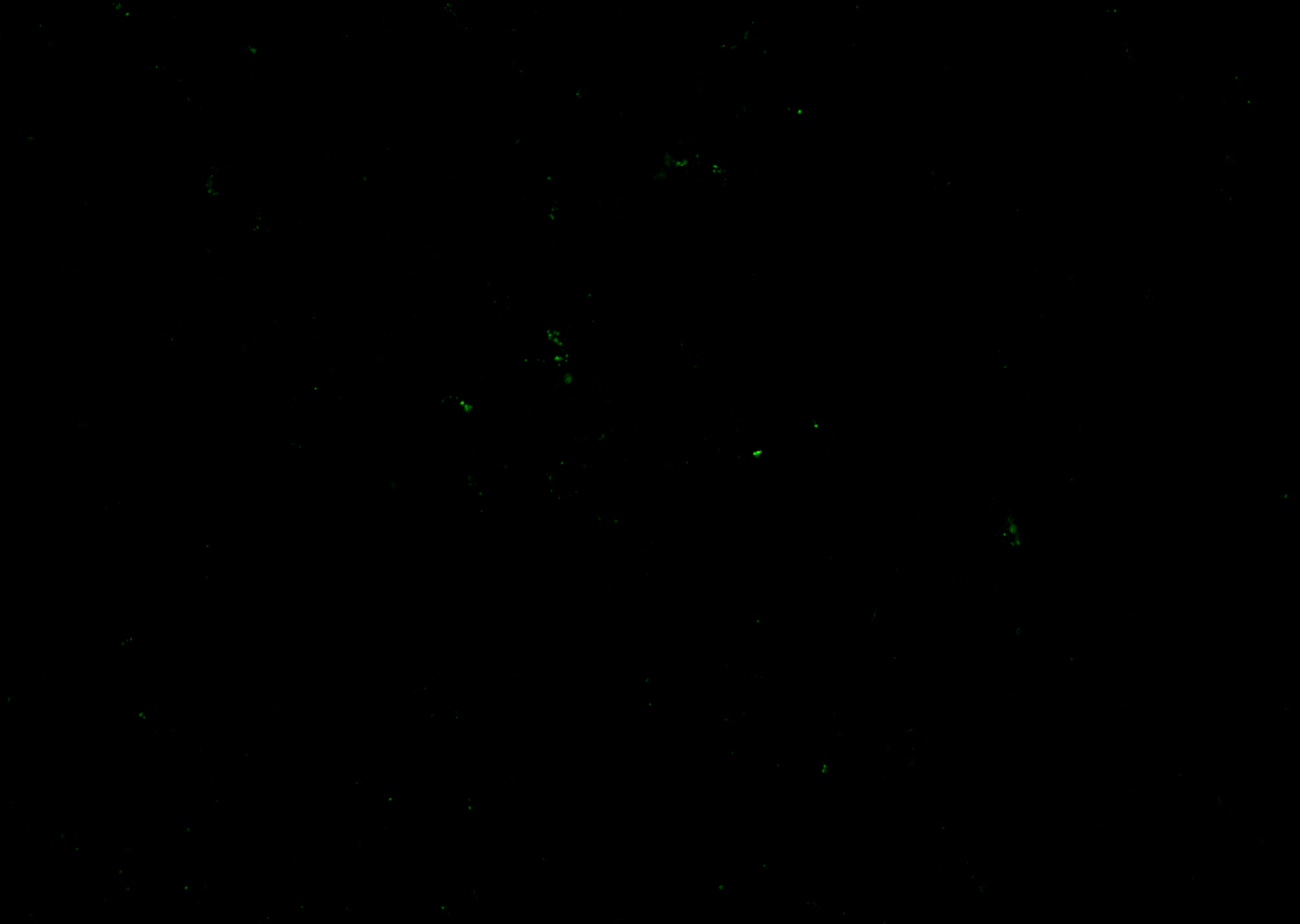

Supplement: Supplementary file 2 [file DataSheet1.zip › Supplementary Materials/MALT lymphoma/20号 CD20红+Aprily8绿 400-2.jpg]

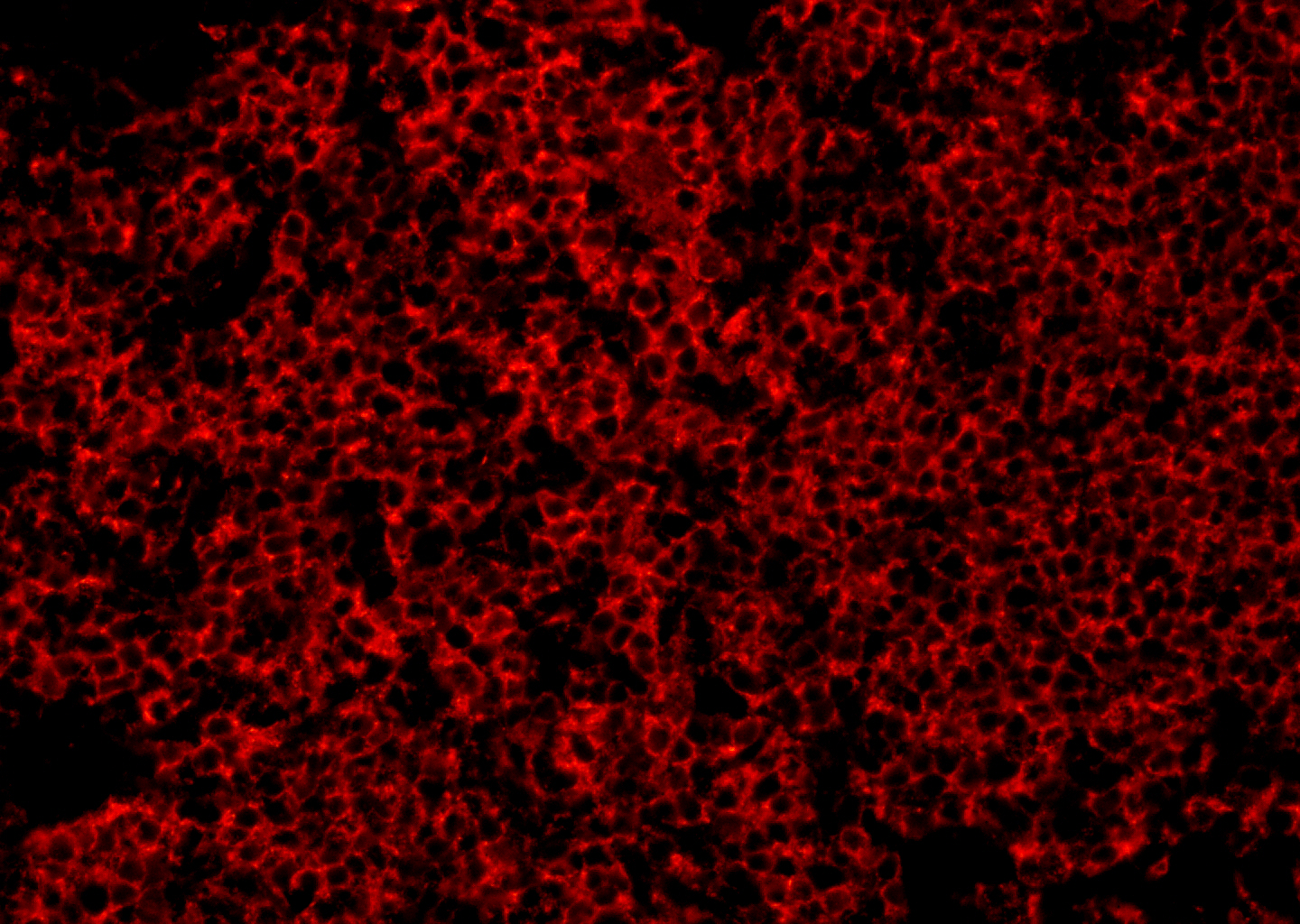

Supplement: Supplementary file 2 [file DataSheet1.zip › Supplementary Materials/MALT lymphoma/20号 CD20红+Aprily8绿 400-4.jpg]

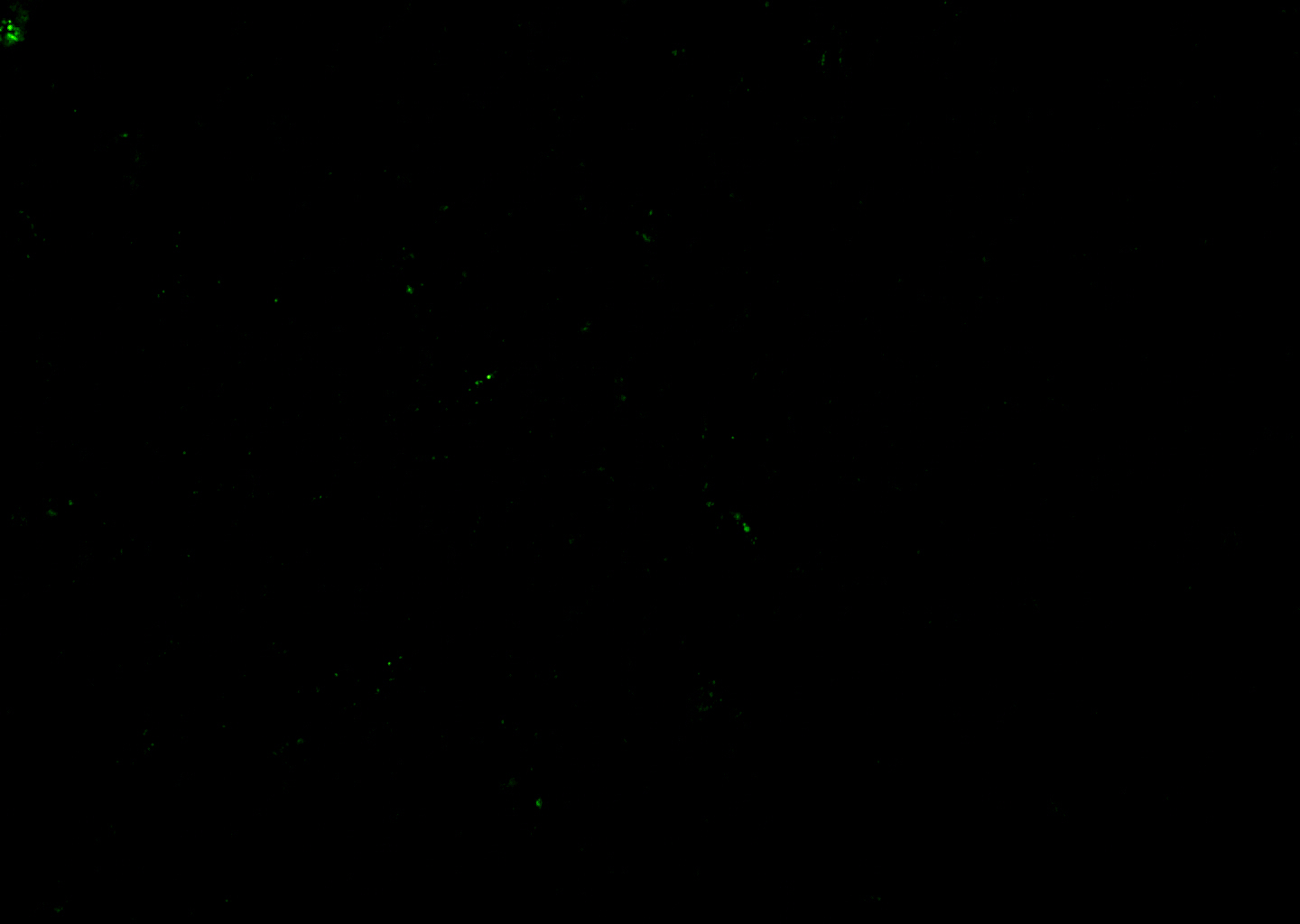

Supplement: Supplementary file 2 [file DataSheet1.zip › Supplementary Materials/MALT lymphoma/20号 CD20红+Aprily8绿 400-5.jpg]

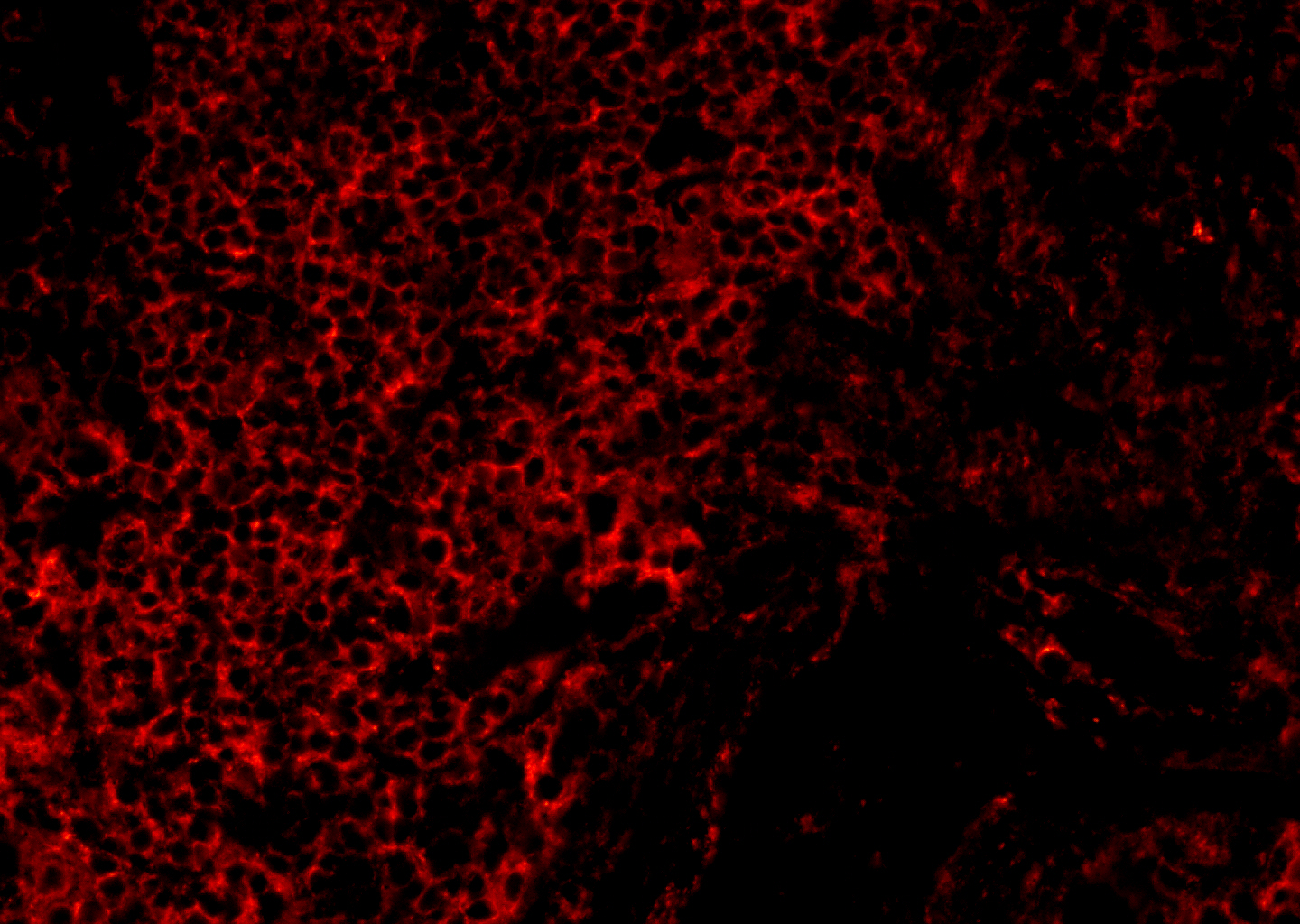

Supplement: Supplementary file 2 [file DataSheet1.zip › Supplementary Materials/MALT lymphoma/20号 CD20红+Aprily8绿 400-7.jpg]

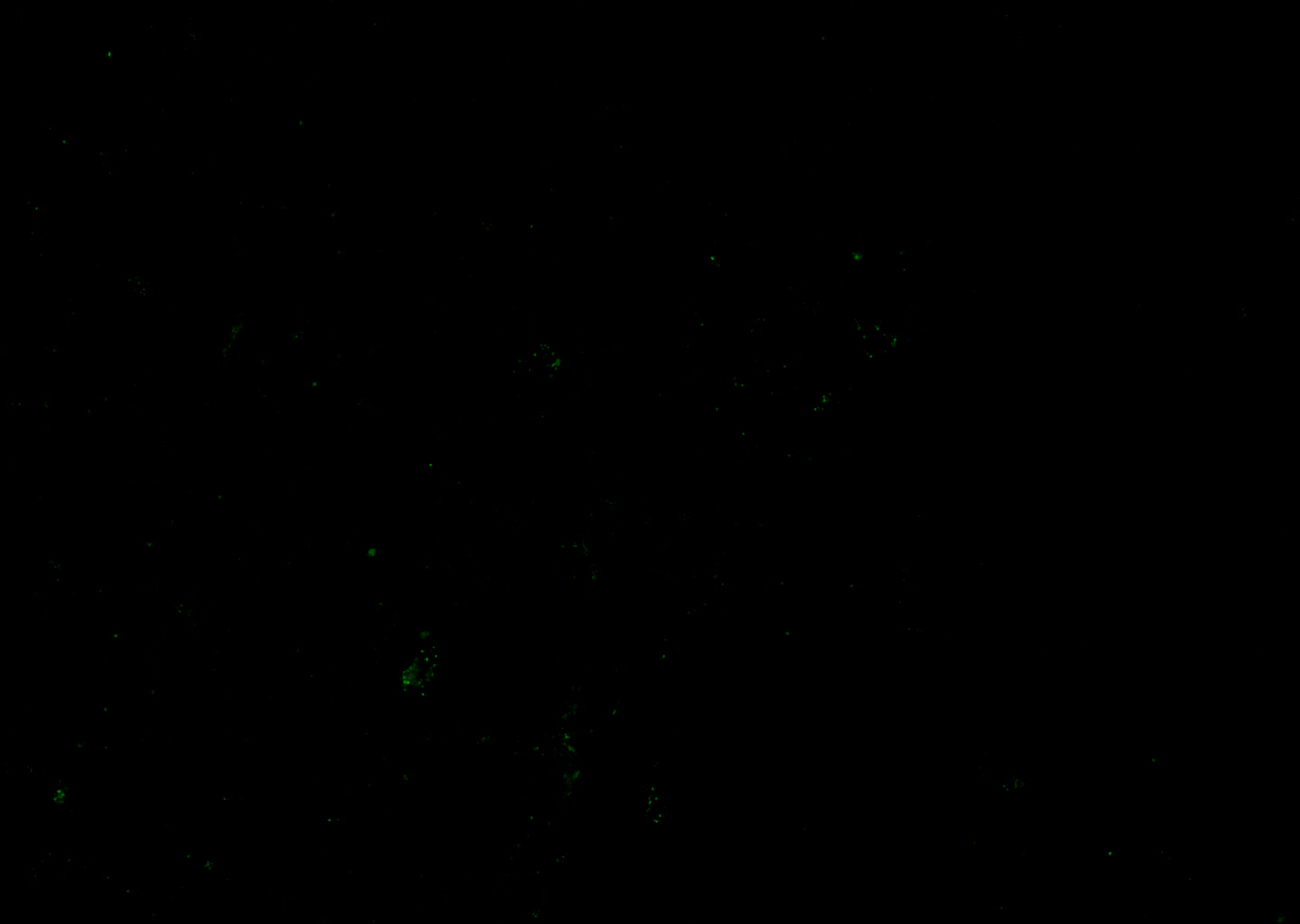

Supplement: Supplementary file 2 [file DataSheet1.zip › Supplementary Materials/MALT lymphoma/20号 CD20红+Aprily8绿 400-8.jpg]
